# Deep learning-based multi-drug synergy prediction model for individually tailored anti-cancer therapies

**DOI:** 10.3389/fphar.2022.1032875

**Published:** 2022-12-15

**Authors:** Shengnan She, Hengwei Chen, Wei Ji, Mengqiu Sun, Jiaxi Cheng, Mengjie Rui, Chunlai Feng

**Affiliations:** Department of Pharmaceutics, School of Pharmacy, Jiangsu University, Zhenjiang, China

**Keywords:** anti-cancer combination therapy, high-order drug combinations, cancer cell subtype-specific models, deep learning framework, precision oncology

## Abstract

While synergistic drug combinations are more effective at fighting tumors with complex pathophysiology, preference compensating mechanisms, and drug resistance, the identification of novel synergistic drug combinations, especially complex higher-order combinations, remains challenging due to the size of combination space. Even though certain computational methods have been used to identify synergistic drug combinations *in lieu* of traditional *in vitro* and *in vivo* screening tests, the majority of previously published work has focused on predicting synergistic drug pairs for specific types of cancer and paid little attention to the sophisticated high-order combinations. The main objective of this study is to develop a deep learning-based approach that integrated multi-omics data to predict novel synergistic multi-drug combinations (DeepMDS) in a given cell line. To develop this approach, we firstly created a dataset comprising of gene expression profiles of cancer cell lines, target information of anti-cancer drugs, and drug response against a large variety of cancer cell lines. Based on the principle of a fully connected feed forward Deep Neural Network, the proposed model was constructed using this dataset, which achieved a high performance with a Mean Square Error (MSE) of 2.50 and a Root Mean Squared Error (RMSE) of 1.58 in the regression task, and gave the best classification accuracy of 0.94, an area under the Receiver Operating Characteristic curve (AUC) of 0.97, a sensitivity of 0.95, and a specificity of 0.93. Furthermore, we utilized three breast cancer cell subtypes (MCF-7, MDA-MD-468 and MDA-MB-231) and one lung cancer cell line A549 to validate the predicted results of our model, showing that the predicted top-ranked multi-drug combinations had superior anti-cancer effects to other combinations, particularly those that were widely used in clinical treatment. Our model has the potential to increase the practicality of expanding the drug combinational space and to leverage its capacity to prioritize the most effective multi-drug combinational therapy for precision oncology applications.

## 1 Introduction

Various carcinogenic factors and pathogenesis have been linked to cancer, which has been identified as a collection of complex diseases (Tolomeo and Simoni, 2002). This complicates the application of a single treatment for a single target, as it activates redundant activities in cancer cells such as various downstream factors and parallel pathways due to compensatory mechanisms (Alexander and Friedl, 2012). Inter-tumor and intra-tumor heterogeneity are a major contributor to drug resistance and disease progression in clinical cancer treatment, ultimately leading to disease relapse (Holohan et al., 2013). Combination therapy has been shown to be a well-established and superior solution to these problems because of its improved clinical efficacy and lack of development of drug resistance. Since the dose of each drug is smaller than what is used in monotherapy, it is possible that the side effects will be minimized (Mahase, 2019). So far, significant efforts have been undertaken to systematically evaluate the synergistic combinations from a large pool of chemical compounds (MacGowan et al., 1990; Wiesner et al., 2002; Sopirala et al., 2010). Finding successful drug combinations is still incredibly difficult, especially with today’s high-throughput screening technologies (Sun et al., 2013). Furthermore, high-order combinations have the potential to regulate biological systems more powerfully than drug pairs because they favor compensatory mechanisms, which tumors greatly exploit; however, the number of experiments run to identify promising high-order combinations would explode by several orders of magnitude, which is far beyond the current exploration ability. There is a pressing need for systemic methodologies, and an urgent need to make it feasible to find new therapeutic combinations of more than two agents, including synthetic chemicals, biological molecules and natural products.

An extensive range of computational methods spanning a large area of methodologies (Bansal et al., 2014; Gayvert et al., 2017; Chen et al., 2018; Huang et al., 2019) has tremendously aided research into anti-cancer drug combinations in the recent years. Different machine learning models and the burgeoning field of deep learning are examples of possible approaches. A machine learning based classification model could extract features from multiple drug profiles including drug targeted proteins and Anatomical Therapeutic Chemical Classification System (ATC) codes, and, as a result, it enabled the prediction of potential synergistic drug pairs (Iwata et al., 2015). But ATC code is available only for marketed drugs, suggesting that the processing of uncharacterized drugs or new candidate compounds is considerably beyond the power of this approach. In another method, two machine learning algorithms, random forest (RF) and extreme gradient boosting (XGBoost), were applied to establish models for drug combination prediction, indicating that XGBoost resulted in a better perform than the RF model (Sidorov et al., 2019). As trained on a pre-cell line, these two models should be rebuilt when applying for another cell line. Recent impressive breakthroughs of deep neural networks, which profit from the explosion of big data and the ability to automatically extract key features, have produced greatly enhanced performance in biomedical research. A deep learning approach, DeepSynergy, proposed by Preuer *et al.*, integrated the chemical descriptors of drugs and genomic data of cell lines of interest for predicting synergistic drug combinations (Preuer et al., 2018). Following this, numerous techniques based on deep learning framework, such as AuDNNsynergy (Zhang T. et al., 2021), MatchMaker (Kuru et al., 2021) and Deep Signaling Synergy (Zhang H. et al., 2021), have been suggested with multi-omics data to prioritize drug combinations, revealing their benefits on the prediction of paired drug combination. However, the existing deep learning models mainly focused on predicting drug pairs which might not be efficient to inhibit the aggressive growth of tumors driven by complex mechanisms (Holohan et al., 2013; Dry et al., 2016).

With the approval of multi-drug combinations for a variety of diseases such as cancers and *tuberculosis* (Gotwals et al., 2017; Davies et al., 2019), the focus of the search for combinational therapies has shifted partially away from pairwise combinations and toward high-order ones containing three or more drugs. Yet there are limited tools to predict multi-drug synergy in diseases. A recent web application, Synergy Finder 2.0, is developed to analyze the drug combination screen data and provide the best multi-drug synergy patterns (Ianevski et al., 2020). However, this tool is based on the dose-response data collected by a huge number of multi-drug screening activities, which make it infeasible to find prospective high-order combinations in a labor- and time-saving manner. So far, we lack deep learning-based approaches to predict the synergy of high-order combinations by integrating multi-omics data, and this is a problem.

The methodological advances of deep learning-based models have made it easier to investigate the best possible high-order combinations within the defined disease module. In this study, we developed a deep learning-based model for the prediction of synergistic multi-drug combinations (DeepMDS) through using a large-scale dataset that integrated by targets information, drug response data and large-scale genomic profile of cancer cell lines from varied tissues. DeepMDS can generate predicted pseudo-IC50 values, which can be used to quantify and, by extension, rank the synergistic anti-cancer effect of drug combinations. As a comparison, we used some of the most advanced machine learning algorithms as reference models, including K Nearest Neighbor (KNN), Random Forest (RF), Support Vector Machine (SVM) and Gradient Boosting Machine (GBM). These algorithms have all been succeeded in modeling drug synergy and were among the top winning methods of the 2019 AstraZeneca-Sanger drug combination prediction DREAM Challenge (Menden et al., 2019). More importantly, the performance of our DeepMDS were further extensively validated by published literatures and rigorous studies based on biologically heterogeneous breast cancer cell subtypes (MCF-7, MDA-MD-468 and MDA-MB-231) as well as lung cancer cell line A549.

## 2 Materials and methods

### 2.1 Data collection

In this work, we collected, pre-processed, and combined gene expression profiles of cancer cell lines and target information of anti-cancer drugs to generate modeling dataset. Then, data on drug response against a large variety of cancer cell lines were also collected for the purpose of labeling modeling samples. Herein, the precise process of datasets construction was described in detail in this section.

#### 2.1.1 Gene expression features

Based on Affymetrix Human Genome U219 Array plates, basal gene expression profiles of 1,000 human cancer cell lines were measured and identified utilizing a wide variety of anti-cancer therapeutics in the Genomics of Drug Sensitivity in *Cancer* (GDSC) project (Iorio et al., 2016). The gene expression data of cancer cell lines were demonstrated to be useful information, which faithfully recapitulated cancer-driven alterations in 11,289 tumors from 29 tissues. Meanwhile, many of the genomic information were highly associated with drug sensitivity or resistance and thus it could be efficiently applied to predict drug response as sample features. The public available transcriptional profiles of 1,000 human cancer cell lines were carefully retrieved from the ArrayExpress database (Parkinson et al., 2005) and then the data pre-processing was conducted based on the platform R v3.5.0. To begin, oligo-package was applied to convert the downloaded raw data (CEL files) into standard genomic profiles. Then missing and invalid values were filled and replaced using the impute 1.52.0 package from Bioconductor Library (Gentleman et al., 2004). In further, Robust Multichip Average (RMA) algorithm was used to normalize the refilled datasets, preventing erroneous results generated by maxima and minima as well as decreasing computing burden. Next, based on the annotation file of gene chip, each probe ID was matched with its corresponding gene symbol and the mean expression value of the multiple probe IDs matched the same official gene symbol was computed to reflect the expression intensity. A phenomenon known as the “curse of dimensionality” may cause prediction models to perform poorly due to the large number of genes covered by the expression profiles (Aliper et al., 2016). To avoid this difficulty, genes in cancer-related pathways were selected to lower the size of gene expression features. In practice, 14 gene sets, which were defined by cBioPortal, consisted of cancer-related pathways (Cerami et al., 2012), such as DNA damage response or RTK signaling pathways (Jeon et al., 2018). Finally, a total number of 215 genes were selected as genomic features and their corresponding gene expression data were used as the feature representations of cancer cell lines (Supplementary Data Sheet S1).

#### 2.1.2 Target information

Along with gene expression features, this study gathered information on the targets of anti-cancer drugs. To begin, we obtained target information for 265 chemical compounds from DrugBank (Wishart et al., 2018) and PubChem (Wang et al., 2009). This information was merged with determined drug sensitivity of cancer cell lines from the GDSC project. On the other hand, 1,574 naturally occurring anticancer compounds were obtained from the Naturally occurring Plant based Anticancerous Compound-Activity-Target DataBase (NPACT), and the related target information for each compound was retrieved from TCMSP (Ru et al., 2014), DrugBank and PubChem. Finally, a total of 1,093 targets were obtained as target features of compounds. The target information of each compound was used to generate the feature representation of the compound. More specifically, the target feature values corresponding to the targets of the compound were encoded as “1” and the others were encoded as “0” (Supplementary Data Sheet S2).

#### 2.1.3 Drug response information

Drug response information, also called as monotherapy information, assessed drug effects on cell lines and was used in this study to label samples. The GDSC project experimentally determined and quantified the drug responses of over 265 chemical compounds to 1,000 cancer cell lines using the half maximum inhibitory concentration (IC50) (Iorio et al., 2016). Additionally, we gathered equivalent data for 1,574 natural chemicals in response to distinct cell lines from NPACT, PubChem and related literatures. In total, the drug responses of 201,405 drug-cancer cell line pairs were collected and used as the labels (IC50 in the regression task and binary value in the classification task) (Supplementary Data Sheet S3).

#### 2.1.4 Data integration

**FIGURE 1:**
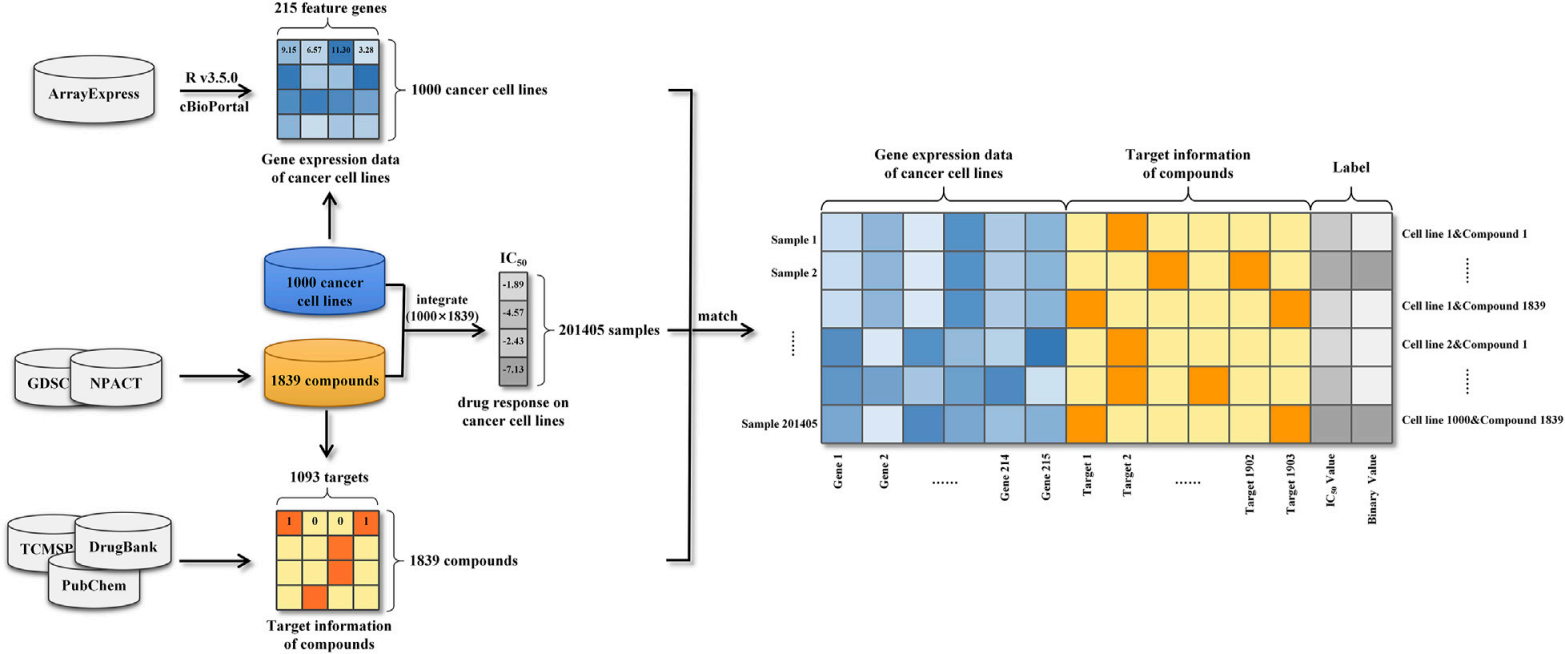
Schematic illustration of the construction of our modeling dataset.

Gene expression profiles of cancer cell lines, target information of anti-cancer compounds and drug responses against a large variety of cancer cell lines were integrated into 201405 modeling samples (Figure 1). Specifically, each sample was represented as a vector consisting of a 215-dimensional genomic feature representation of cancer cell line and a 1093-dimensional target feature representation of compound. Following that, the drug response was used to label the sample. Due to the considerable dimension disparity between gene expression features and target information, all samples’ data were adjusted using zero-centered processing and normalized square deviation.

### 2.2 Model construction

Among the processed datasets, 80% (161,124) of samples were randomly chosen for the training dataset, while 20% (40,281) were used as the test dataset. Then, using the training and test datasets, a deep learning prediction model and other models based on various machine learning algorithms were constructed and optimized, and their performances were compared.

#### 2.2.1 Deep learning prediction model

**FIGURE 2:**
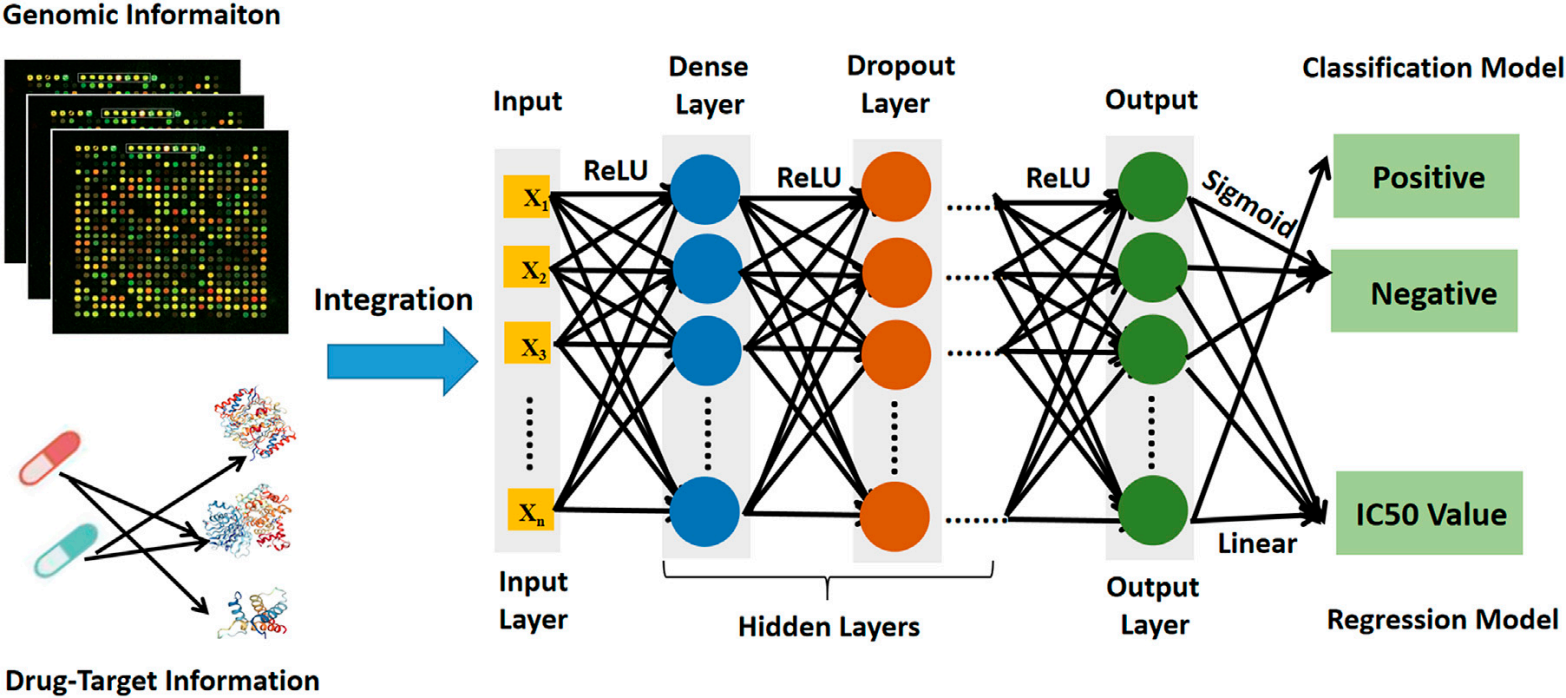
The architecture diagram of deep learning prediction model showing data sources and workflow.

The deep learning prediction model (DeepMDS) was built sequentially in Python (version 3.6) using the Keras platform, which is a high-level neural networks API running on top of Theano (Feng et al., 2019). The basic architecture of deep learning models was illustrated in Figure 2. To begin, gene expression data from cell lines and target information of drugs as input were loaded in the nodes (also called neurons) of the input layer. Then the loaded information from input layer was propagated through the neighboring hidden layers, including the dense layer and the dropout layer. Finally, the output layer could provide the predicted IC50 values for each sample. To address sophisticated regression problems, each layer among the deep learning architecture was followed by non-linear activation functions (Feng et al., 2019). The Rectified Linear Unit (ReLU) activation function was used to activate the input layer and hidden layer in this study because it has the capacity to reduce the vanishing gradient problem and has a rapid computing speed (Eq. 1) (Feng et al., 2019). Then for the output layer, a linear activation function was applied in the regression model to fit the distribution of predicted IC50 values better (Eq. 2). Meanwhile, the classification model was developed using the deep learning architecture, which enables a similar assessment of model performance. The construction of classification model constructed in the same manner as stated previously, except that the Sigmoid activation function (Eq. 3) was applied to produce the classification labels in the output layer (Eq. 3). Here, the samples labeled with IC50 values ≤10 nM were considered positive samples, whereas those labeled with IC50 values >10 nM were considered negative samples.

$$y=ReLU\left(Wx+b\right)$$ (1)

where *y* was the activation value of the hidden layer, *x* was the input data, *W* was weight matrix and b was bias.

$$z=linear\left(W^{\prime }y+b\prime \right)$$ (2)

where *z* was the predicted IC50 values, *y* was the activation value of the hidden layer, *W′* was transposed weight matrix and *b'* was transposed bias.

$$z=sigmoid\left(W^{\prime }y+b\prime \right)$$ (3)

where *z* was the classification labels, *y* was the activation value of the hidden layer, *W′* was transposed weight matrix and *b'* was transposed bias.

In order to train the model, the loss functions of MSE (mean square error) and binary cross-entropy were used to estimate performance of regression and classification models, respectively, by comparing the difference between the actual label of input data in input layer (*x*) and the predicted label of output layer (*z*), where SGD (stochastic gradient descent) was applied to search the optimal parameters (Eq. 4).

$$L_{H}\left(x,z\right)=-\overset{d}{\underset{k=1}{\sum }}\left[x_{k}\log z_{k}+\left(1-x_{k}\right)\log\left(1-z_{k}\right)\right]$$ (4)

where *x* was the actual value of input data in input layer, *z* was the predicted value of output layer, *d* was the epoch number.

In addition, Adam (adaptive moment estimation) and RMSprop (Root Mean Square prop) were selected as optimization functions for the construction of regression and classification models, respectively. Throughout the training process, the aforementioned processes were repeated in order to update the weights and bias until the optimal weight matrix *W* and bias *b* were obtained.

##### 2.2.1.1 Optimization of deep learning prediction model

The performance of a deep learning prediction model is determined not only by its architecture of deep learning but also by its hyper parameters. Traditionally, the ideal parameter combination for a deep learning model was established by human experience, which was neither accurate nor objective. To obtain the optimal DeepMDS, a grid search algorithm was used to find the best combination from a parameter space including epoch number, batch size, learning rate, dropout rate and hidden units of hidden layers. Finally, using the same datasets, 5,625 (5 × 5×3 × 3 × 5 × 5) regression and classification models were developed individually to seek their own optimal parameter combinations using 10-fold cross validation. According to the optimization results, the conic architecture with two hidden layers having 200 nodes in the first layer and 100 nodes in the second layer was the optimal regression and classification model.

Also, a big dropout rate of 0.5 followed behind each dense layer to avoid the overfitting problems. Furthermore, a smaller learning rate of 10^−5^, a batch size of 128 and an epoch number of 200 were set up to constitute the optimal regression model. Meanwhile, a learning rate of 10^–3^, a batch size of 32 and an epoch number of 500 were chosen for the best classification model (Supplementary Table S2, Supplementary Data Sheet D4).

#### 2.2.2 Model evaluation and comparison

To compare the performance of deep learning model to that of other models based on state-of-the-art machine learning algorithms, the same datasets were used to develop a k nearest neighbor (KNN) model, a random forest (RF) model, a support vector machine (SVM) model and a gradient boosting machine (GBM) model. Also, each model was allowed to optimize hyper parameters using a grid search algorithm and cross validation.

##### 2.2.2.1 K nearest neighbor model

The variable selection k nearest neighbor (KNN) algorithm was applied to develop the prediction model based on Python (version 3.6). Regarding hyper parameter setting, number of neighbors, types of weight functions and algorithms were tuned to achieve the optimal KNN model. Following a grid search in a value space of considered parameters, the optimal parameters for the KNN regression model were 6 neighbors, a “uniform” weight function and a ‘auto’ algorithm. In addition, for the KNN classification model, the optimal model consisted of 5 neighbors, a “uniform” weight function, and a “auto” algorithm (Supplementary Table S3, Supplementary Data Sheet S4).

##### 2.2.2.2 Random forests model

Based on random forest (RF) algorithm and Bagging architecture, Random Forest Regressor and Random Forest Classifier functions were used to develop RF regression and classification models using Python (version 3.6) respectively. In terms of hyper parameter setting, the number of features considered in each split, the number of estimators (trees), and the minimal number of leaved samples were all adjusted. As a consequence, the RF regression model’s optimized parameters were 200 estimators, ‘auto’ for features considered, and a min_samples_leaf of 50. The best settings for the RF classification model were set at 100 estimators, ‘auto’ for features considered, and a min_samples_leaf of 10 (Supplementary Table S4, Supplementary Data Sheet S4).

##### 2.2.2.3 Support vector machine model

Based on Support Vector Machine (SVM) algorithm, Support Vector Regression (SVR) and Support Vector Classification (SVC) functions were applied to develop SVM regression and classification models using Python (version 3.6) respectively. During the process of hyper parameter setting, the type of kernel function, penalty factor C and gamma were tuned to achieve the optimal SVM model. According to the optimization results, the optimal SVM regression model was determined to be the RBF kernel function, a penalty factor C of 10 and a gamma of 0.01. Then, for the SVM classification model, the optimal parameters were determined to be the RBF kernel function, a penalty factor C of 1 and a gamma of 0.1 (Supplementary Material S5, Supplementary Data Sheet S4).

##### 2.2.2.4 Gradient boosting machine model

Based on Gradient Boosting Machine (GBM) algorithm and Boosting architecture, Gradient Boosting Regressor and Gradient Boosting Classifier functions were applied to construct GBM regression and classification models *via* Python (version 3.6), respectively. When setting the hyper parameters, number of trees, learning rates, number of features in each split, min_ samples_ split and min_ samples_ leaf were took into consideration. According to the optimization results, the optimal GBM regression model consisted of 500 estimators, a min_ samples_ split of 1,000, a learning rate of 0.01, and a min_ samples_ leaf of 60. Also, the optimal parameters for the GBM classification model were then adjusted as 200 estimators, a min_ samples_ split of 600, a learning rate of 0.01, and a min_ samples_ leaf of 60 (Supplementary Material Table S6, Supplementary Material Data D4).

#### 2.2.3 Performance metrics

In order to assess and compare the performances of above optimized prediction models, the mean square error (MSE, Eq. 5), the root mean square error (RMSE, Eq. 6) and R-Square (*R* ^2^_score, Eq. 7) were used as metrics to evaluate their ability to predict IC50 values of drug combinations in the regression task. Meanwhile, the standard criteria for classification work including Sensitivity (SEN, Eq. 8), Specificity (SPE, Eq. 9), Accuracy (ACC, Eq. 10) and Matthews correlation coefficient (MCC, Eq. 11) were also applied to evaluate model performance for the classification task.

$$MSE=\frac{1}{m}{\overset{m}{\underset{i=1}{\sum }}\left(y_{true}^{\left(i\right)}-y_{pre}^{\left(i\right)}\right)}^{2}$$ (5)

$$RMSE=\sqrt{\frac{1}{m}\overset{m}{\underset{i=1}{\sum }}{\left(y_{true}^{\left(i\right)}-y_{pre}^{\left(i\right)}\right)}^{2}}$$ (6)

$$R^{2}\_score=1-\frac{\frac{1}{m}\overset{m}{\underset{i=1}{\sum }}{\left(y_{true}^{\left(i\right)}-y_{pre}^{\left(i\right)}\right)}^{2}}{\frac{1}{m}\overset{m}{\underset{i=1}{\sum }}{\left(y_{true}^{\left(i\right)}-\overset{-}{y}\right)}^{2}}$$ (7)

where *y*_*true*_ was the actual values of samples, *y*_*pre*_ was the predicted values of samples, *m* was the number of samples.

$$SEN=\frac{TP}{TP+FN}$$ (8)

$$SPE=\frac{TN}{FP+TN}$$ (9)

$$ACC=\frac{TP+TN}{TP+FN+FP+TN}$$ (10)

$$MCC=\frac{TP*TN-FP*FN}{\sqrt{\left(TP+FN\right)*\left(TP+FP\right)*\left(TN+FN\right)*\left(TN+FP\right)}}$$ (11)

where TP meant true positive; TN meant true negative; FP meant false positive; FN meant false negative.

Furthermore, the area under the Receiver Operating Characteristic (ROC) curve (AUC) was also used to evaluate the model performance for the classification task. Specifically, the best possible prediction was 100% sensitivity and 100% specificity with area under the curve (AUC) of 1, while an AUC value of ≤0.5 represented random selection.

### 2.2 Prediction and validation with literature synergy data

To further verify the performance of constructed DeepMDS model built above, literature validation was carried out. Sun’s work (Sun et al., 2015) rated 17 drug pairs comprised of 12 single agents (sorafenib, erlotinib, gefitinib, tamoxifen, everolimus, dasatinib, sunitinib, BIBW-2992, thalidomide, PD98059, flavopiridol and toremifene) based on their RACS model-predicted synergy. Meanwhile, to confirm the predicted results, each drug pair was experimentally tested at four different concentration ratios (4:1, 3:2, 2:3, and 1:4) using MCF-7 cell line. The synergistic effect of these 17 drug pairs was also predicted and ranked by DeepMDS using target information and gene expression data of MCF-7 cell line. DeepMDS’s predicted results were then compared to experimental results from the literature to determine the model’s performance.

### 2.3 Prediction and validation by *in vitro* cellular experiments

To further evaluate the capability of DeepMDS to predict the synergy effect of multi-drug combinations, seven recommended chemotherapy drugs (docetaxel, paclitaxel, doxorubicin, epirubicin, gemcitabine, 5-fluorouracil, and methotrexate) from breast cancer clinical treatment guidelines (Telli and Carlson, 2009) were randomly grouped to generate drug combinations, including drug pairs and high-order combinations. Following that, the synergy effect of drug combinations was then predicted using DeepMDS and evaluated using an *in vitro* cell viability assay. In brief, 120 drug combinations were constructed using seven chemotherapeutic agents (II2-II28 indicated two-drug combinations, III10-III56 indicated three-drug combinations, Ⅳ16–Ⅳ70 indicated four-drug combinations, Ⅴ21–Ⅴ56 indicated five-drug combinations, Ⅵ16–Ⅵ28 indicated six-drug combinations, Ⅶ7 indicated seven-drug combinations).

Following the collection of target information for each medication from GDSC, PubChem, and DrugBank, the datasets were pre-processed to construct prediction samples. To examine the synergistic effect of the aforementioned drug combinations, three distinct subtypes of breast cancer cell lines were used: MCF-7, MDA-MB-468, and MDA-MB-231. Furthermore, to validate DeepMDS’s robustness and applicability, this model was used to predict another cancer cell line A549 from lung tissue. Each cell line’s gene expression data were analyzed and then utilized to construct prediction samples. Finally, DeepMDS was used to predict the sample datasets. For each cell line, the optimized DeepMDS model predicted and ranked the IC50 values of 120 drug combinations.

The corresponding validation experiments were carried out *in vitro*. MCF-7, MDA-MB-468, MDA-MB-231, and A549 cell lines were obtained from the Cell Bank of Type Culture Collection of Chinese Academy of Sciences (CBTCCCAS). Four cancer cells were cultured in DMEM medium supplemented with 10% fetal bovine serum, and kept at 37°C and 5% CO_2_ in a humidified incubator. Docetaxel, paclitaxel, doxorubicin, epirubicin, gemcitabine, 5-Fluorouracil and methotrexate were purchased from Meryer (Shanghai, China), and the purity of each drug (compound) is above 98%. Each drug (compound) was dissolved in DMEM medium and then used alone or in combination with other drugs at various concentration ratios so that we could ensure each drug attained its best synergistical ratio throughout a wide concentration range (Table 1). Then, exponentially growing cells were seeded in 96-well plates at a density of 5×10^3^ per well and cultured for 24 h.

**TABLE 1:** The settings of concentration ratios for different drug combinations.

The number of drugs in a combination	Ⅰ	Ⅱ	Ⅲ	Ⅳ	Ⅴ	Ⅵ
Two	1:1	2:1	1:2	—	—	—
Three	1:1:1	2:1:1	1:2:1	1:1:2	—	—
Four	1:1:1:1	2:1:1:1	1:2:1:1	1:1:2:1	1:1:1:2	—
Five	1:1:1:1:1	2:1:1:1:1	1:2:1:1:1	1:1:2:1:1	1:1:1:2:1	1:1:1:1:2

Note: roman numerals, including Ⅰ, Ⅱ, Ⅲ, Ⅳ, Ⅴ, and Ⅵ, indicated different drug molar ratios in a drug combination.

Afterward, the cells were then treated for 72 h with a variety of single drugs or multi-drug combinations at a series of diluted concentrations. There are three replicates for each measurement, and the cytotoxicities of individual drugs or combinations were determined using the cell counting kit-8 (CCK-8) assay. IC50 values for each sample was calculated in line with the manufacturer’s instructions. In addition, the combination index (CI) (Chou and Talalay, 1984) was calculated using the CompuSyn software (Chou and Martin, 2007), and then CI values were applied to define and quantify the synergistic effect of each drug combination. In general, a drug combination is synergistic if the CI value is less than 0.9, additive if the CI value is between 0.9 and 1.1, and antagonistic if the CI value is greater than 1.1 (Sun et al., 2016). In this study, a drug combination was considered synergistic if the CI values for all concentration ratios were all less than 0.9.

### 2.4 Pathway enrichment analysis of drug combinations

To explore the synergistic mechanism of predicted combinations in given cell lines, KEGG pathway enrichment analysis was performed on the specific feature genes of cancer cell lines and the target information of drug combinations, and the pathways of synergistic combinations against different cancer cell lines were compared.

## 3 Results

### 3.1 Overview of DeepMDS model

Here, we present DeepMDS, a Deep Neural Network (DNN)-based methodology for the prediction of the pseudo-IC50 values of a series of drug combinations in a given cell line. Figure 2 illustrates the framework of the DeepMDS, which contains two main features: 1) identification of top-ranked drug combinations from a pool of drug pairs and combinations of three or more compounds, that is, high-order combinations, and 2) cancer cell line-specific prediction by integrating gene expression profile, target information of drugs, and drug responses. In other words, DeepMDS not only allows us to predict the most potent combination, but it also allows us to deliver the best prospective combination susceptible to a specific molecular subtype of cancer cells, which mimics the way that precision medicine is utilized in clinical trials.

### 3.2 Model comparison

We first validated our DeepMDS using the test dataset and compared it to four other machine learning-based methods (Table 2 and Table 3). In terms of performance metrics, regardless of whether the regression task is used to predict he pseudo-IC50 values or the classification task is used to identify positive results, it is clear that our deep learning model outperformed those developed using traditional machine learning algorithms. In specific, DeepMDS achieved a test MSE of 2.50 in the regression task, while GBM, SVM, RF and KNN models performed poorly with MSEs of 5.75, 8.66, 13.11 and 16.73, respectively. Along with MSE, two more evaluation metrics, RMSE and *R* ^2^_score, showed a similar trend. It is worth mentioning that the square root of *R* ^2^_score equals the Pearson correlation coefficient in this case, as *R* ^2^_score was used to determine the linear correlation between predicted and actual values in this regression task. In the classification challenge, DeepMDS also outperformed the competition, increasing the ACC to 0.94 and the AUC to 0.97, while the second-best approach, the GBM model, achieved an ACC of 0.86 and an AUC of 0.92.

**TABLE 2:** Model performances of prediction models for regression task.

Model	MSE	RMSE	*R* ^2^_score
DeepMDS	2.50	1.58	0.86
GBM	5.75	2.40	0.81
SVM	8.66	2.94	0.75
RF	13.11	3.62	0.72
KNN	16.73	4.09	0.67
DeepSynergy	255.49	15.91	0.73

Note: The columns showed mean square error (MSE), root mean square error (RMSE) and R-Square (*R* ^2^_score).

**TABLE 3:** Model performances of prediction models for classification task.

Model	SEN	SPE	MCC	ACC	AUC
DeepMDS	0.95	0.93	0.88	0.94	0.97
GBM	0.87	0.85	0.72	0.86	0.92
SVM	0.81	0.85	0.66	0.83	0.89
RF	0.74	0.82	0.56	0.78	0.83
KNN	0.75	0.71	0.46	0.73	0.76
DeepSynergy	0.57	0.95	NA	0.92	0.90

Note: The columns showed sensitivity (SEN), specificity (SPE), Matthews correlation coefficient (MCC), accuracy (ACC), and the performance measures area under ROC, curve (AUC). “NA” indicated that no MCC, data was provided in literature.

Additionally, we compared the performance of DeepMDS to that of DeepSynergy, a deep learning-based model for predicting synergy in a given cell line. DeepSynergy achieved an ACC of 0.92 and an AUC of 0.90 for classification, and an MSE of 255.49 and an RMSE of 15.91 for regression. As shown in Table 2 and 3, our DeepMDS still performed well. Also, DeepMDS achieved a SEN of 0.95 and a SPE of 0.93 for the classification task, compared to 0.57 and 0.95 for DeepSynergy. Moreover, we compared the performance of DeepMDS against other deep learning-based methods. DeepMDS predictions showed a significant correlation with actual combination viabilities (Pearson’s *r* = 0.93, Supplementary Table S1), outperforming other four models developed in the last 2 years. These findings demonstrated that the strength of our deep learning-based model, which was able to achieve steady and robust model performance in both regression and classification tasks, as well as superior accuracy in drug synergy prediction.

### 3.3 Literature validation

**FIGURE 3:**
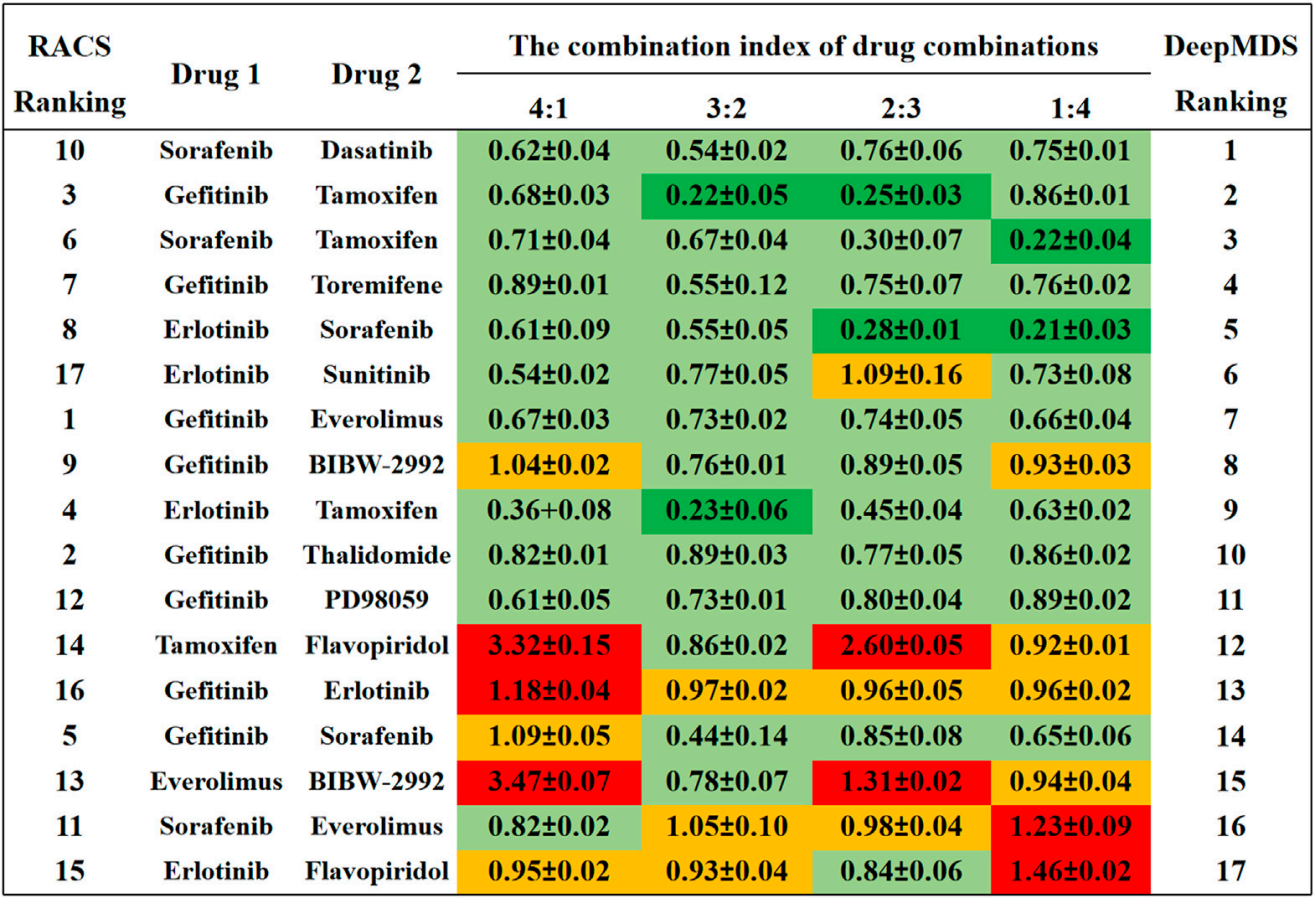
The comparison results between DeepMDS and literature. The synergy effect of each drug pair was retrieved from literature (Sun et al., 2015), and described using combination index (CI). The left ranking was predicted using RACS model, validated by *in vitro* experiments on MCF-7 (Sun et al., 2015). Dark green indicated strong synergy (CI < 0.3); pale green indicated synergy (0.3 < CI < 0.9); yellow indicated additive (0.9 < CI < 1.1); and red indicated antagonism (CI > 1.1). The different CI values of each drug pair were calculated at four dual-drug ratios, including 4:1, 3:2, 2:3, and 1:4.

To verify our DeepMDS’s predictive power, we first focused on previously published drug combinations, the majority of which were paired combinations. Seventeen drug pairs and twelve single agents were predicted using DeepMDS and were shown to be consistent with published literature (Sun et al., 2015) (Supplementary Data Sheet S5). Notably, the output layer of the DeepMDS was the predicted pseudo-IC50 value for each combination, which did not represent the actual therapeutic efficacy but was used to rank the therapeutic efficacies of multi-drug combinations. Here we confined the predicted outcomes to pairwise drug combinations and ranked 17 drug pairs according to their increasing pseudo-IC50 values, followed by a comparison to experimental data from the literature (Figure 3).

Four of the seventeen drug pairs in the reference data were validated as having significant synergistic antitumor effects at optimal dose ratios (Sun et al., 2015), as seen by the dark green coloration in Figure 3. DeepMDS re-ranked these drug combinations, revealing that three highly synergistic couples were correctly predicted in the top five combinations. Sorafenib and dasatinib and gefitinib and toremifene, the next two most effective medication combinations, also revealed synergistic mechanisms at all drug ratios. Notably, the bottom-ranked combination was verified to exhibit additive or even antagonistic effects, as predicted by DeepMDS. Collectively, the ranking of pairwise combinations predicted by DeepMDS was largely comparable with experimental data from the literature (Sun et al., 2015), demonstrating our model’s adeptness at filtering and enriching synergistic medication combinations.

### 3.4 *De novo* prediction of multi-drug combinations for specific cancer cell lines

To further explore DeepMDS’s ability to predict novel high-order combinations, we chose seven anticancer drugs that have been approved by the FDA for breast cancer (National Comprehensive Cancer Network, 2021). These drugs were randomly assigned into 120 combinations, ranging from simple drug pairs to more sophisticated three- or more-drug combinations. The anticancer activity of these combinations was then predicted using our DeepMDS on four cancer cell lines, followed by in-house experimental validation.

Regarding the heterogeneous biological markers of breast cancer cell lines, we chose three representative subtypes: MCF-7 for luminal A subtype (ER^+^, PR^+/−^, HER2^−^), MDA-MB-468 for basal subtype (ER^−^, PR^−^, HER2^−^), and MDA-MB-231 for claudin-low subtype (ER^−^, PR^−^, HER2^−^), the latter two of which were also referred to as triple-negative cell lines (Holliday and Speirs, 2011). For the sake of comparison, one lung cancer cell line A549 was chosen to assess the prediction ability of DeepMDS. Then, 120 drug combinations were ranked according to their predicted pseudo-IC50 values for each cell line.

According to the findings (Table 4), the top three synergistic combinations for MCF-7 and MDA-MB-468 shared commonalities, including III12 and III7. When compared to MDA-MB-468, the top three choices for another triple-negative MDA-MB-231 had no similar result. The top three regimens for MDA-MB-231 were combinations of more than three drugs, including Ⅳ33, Ⅴ32 and Ⅳ59. On another A549 lung cancer cell line, Ⅱ28, Ⅲ12 and Ⅲ52 were the top three.

**TABLE 4:** The top three predicted combinations for a variety of cancer cell lines.

Predicted ranking	MCF-7	MDA-MB-468	MDA-MB-231	A549
1	III12 (doxorubicin, docetaxel, and gemcitabine)	Ⅲ12 (doxorubicin, docetaxel and gemcitabine)	Ⅳ33 (doxorubicin, gemcitabine, methotrexate, and paclitaxel)	Ⅱ28 (epirubicin and paclitaxel)
2	Ⅲ7 (doxorubicin, 5-Fluorouracil, and docetaxel)	Ⅱ3 (doxorubicin and docetaxel)	Ⅴ32 (doxorubicin, docetaxel, gemcitabine, methotrexate, and paclitaxel)	Ⅲ12 (doxorubicin, epirubicin, and paclitaxel)
3	Ⅲ18 (doxorubicin, gemcitabine, and paclitaxel)	Ⅲ7 (doxorubicin, 5-Fluorouracil and docetaxel)	Ⅳ59 (5-Fluorouracil, docetaxel, methotrexate and epirubicin)	Ⅲ52 (docetaxel, epirubicin, and paclitaxel)

### 3.5 Experimental validation of predicted synergistic combinations

**FIGURE 4:**
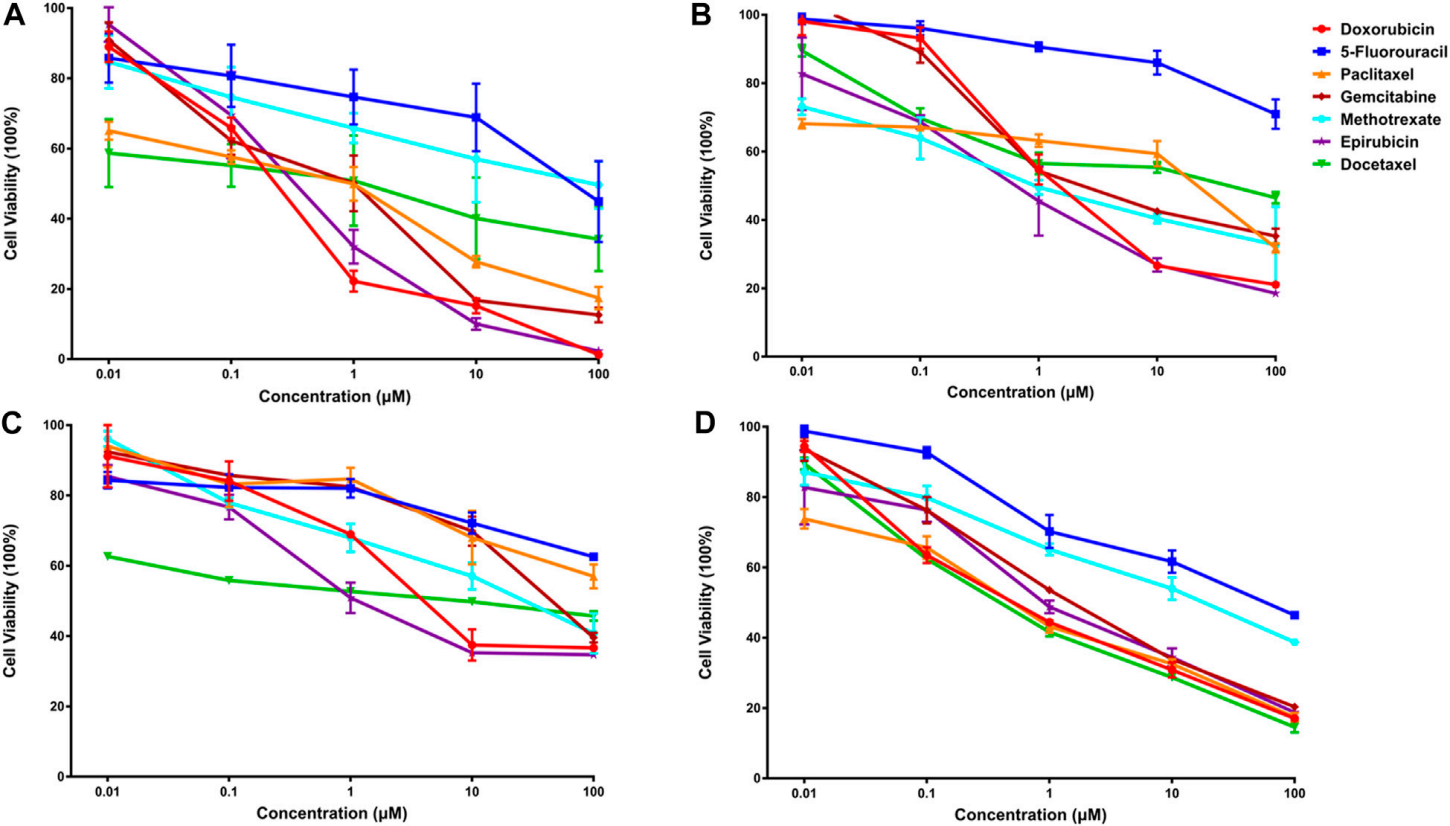
The anti-cancer effects of seven single drugs on four cancer cell lines **(A)**. The anti-cancer effects of seven single drugs on three breast cancer lines, including luminal A subtype MCF-7 **(A)**, basal subtype MDA-MB-468 **(B)**, and claudin-low subtype MDA-MB-231 **(C)**. Also, the anti-tumor ability of seven individual drugs were examined on a lung cancer cell A549 **(D)**.

Subsequently, an *in vitro* cell viability study was undertaken on each cancer cell line to evaluate the predicted findings. IC50 values for individual drugs were first obtained for each cancer cell line (Figure 4). Then, in a similar fashion, the synergistic effects of predicted drug combinations were measured for each cell line.

#### 3.5.1 Synergistic effects of predicted combinations on luminal a breast cancer cell line

For MCF-7 cell line, the results indicated that realistic IC50 values for single drugs ranged from 138.3 nM to 97.25 μM, with docetaxel exhibiting the best anti-cancer ability and 5-fluorouracil exhibiting the least (Figure 4A). In light of the ranked combinations, several combinations containing the top three (Ⅲ12, Ⅲ7 and Ⅲ18), the middle level ones (Ⅲ40, Ⅲ43 and Ⅲ47), and the bottom three (Ⅲ9, Ⅲ44 and Ⅲ41) were examined on MCF-7 cell line using the defined drug ratios listed in Table 1.

As a result, the lowest IC50 value for each combination across all drug ratios was considered the best experimental result and was used to rank the synergistic effect (Table 5). Except for the bottom combinations Ⅲ9 and Ⅲ44, the rest of experimental results were identical to the predicted order. With respect to the combinations Ⅲ9 and Ⅲ44, the actual IC50 values reversed their ranking, which could be explained in part by the fact that both combinations elicited strong antagonistic responses on MCF-7 cell line, and DeepMDS may be insensitive to negative examples with additive or antagonistic effects. Additionally, the associated CI values of drug combinations were also calculated (Supplementary Figure S1). The top three combinations had a clear synergy impact on MCF-7 cell line (0.3 < CI < 0.9), with Ⅲ12 exhibiting the strongest synergy effect (CI < 0.3). By contrast, the middle three and the bottom three demonstrated antagonism effect (CI > 1.1).

**TABLE 5:** The anti-cancer effects of nine combinations of three drugs on MCF-7 cells.

Predicted ranking	Group number	The IC50 (nM) of drug combinations				The best IC50
		Ⅰ	Ⅱ	Ⅲ	Ⅳ	
1	Ⅲ12	90.43	31.37	30.88	42.94	30.88
2	Ⅲ7	290.99	101.66	56.52	85.66	56.52
3	Ⅲ18	102.14	128.37	88.62	168.34	88.62
62	Ⅲ40	2268.13	535.42	5823.46	308.97	308.97
63	Ⅲ43	640.90	6386.59	4144.85	598.10	598.10
65	Ⅲ47	10584.30	827.17	18532.30	10219.80	827.17
118	Ⅲ9	23145.90	11,675.80	2121.31	2784.08	2121.31
119	Ⅲ44	2606.11	2197.47	1499.70	1523.97	1499.70
125	Ⅲ41	6022.00	354825.00	84020.00	24170.00	6022.00

Note: the predicted ranking included 120 drug combinations and individual drugs themselves.

**FIGURE 5:**
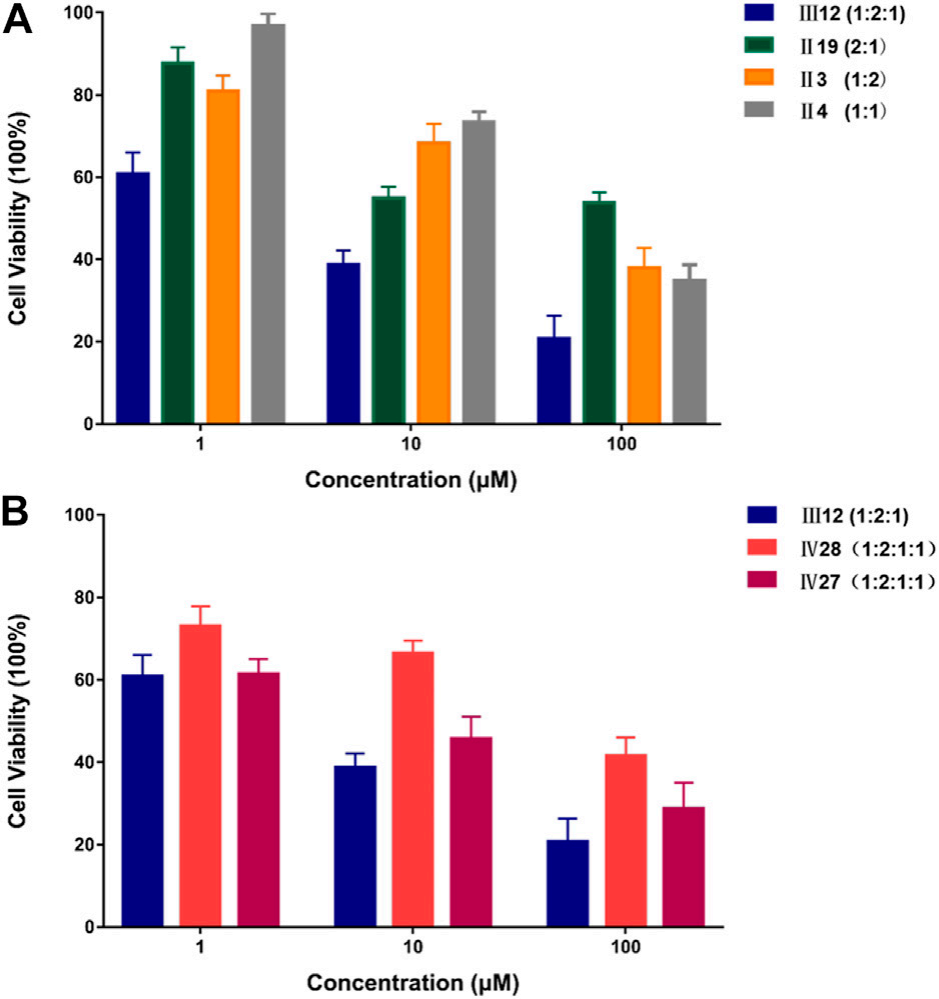
The comparison of anti-cancer effect on MCF-7 cell line between III12 and related combinations. **(A)**. The comparison of anti-cancer effect on MCF-7 cells between III12 and related pairwise combinations that were extracted from III12. **(B)**. The comparison of anti-cancer effect on MCF-7 cells between III12 and four-drug combinations which included the entire III12 composition.

To further evaluate DeepMDS’s accuracy and robustness, the best synergistic combination, III12, was compared to different combinations including either two or all three drugs from III12 at the optimal drug ratio. For example, we chose Ⅱ19 (docetaxel/gemcitabine, 2:1), Ⅱ3 (doxorubicin/docetaxel, 1:2), and Ⅱ4 (doxorubicin/gemcitabine, 1:1) as components of III12 (Figure 5A); and other groups, IV27 and IV28, contained the whole combination setting of III12 (Figure 5B). In addition, the commonly used clinical combinations (Ⅲ55: gemcitabine, epirubicin and paclitaxel, and Ⅱ3: doxorubicin and docetaxel) (Telli and Carlson, 2009) were assessed under the same circumstance as combination Ⅲ12. Not unexpectedly, *in vitro* cellular experimental results indicated that Ⅲ12 continues to exhibit the best anti-cancer synergistic activity when compared to any other combination (Table 6 and Supplementary Figure S2). Taking all the above validation data into account, the predicted Ⅲ12 (doxorubicin, docetaxel and gemcitabine) was the most synergistic combination for the MCF-7 cell line.

**TABLE 6:** The comparison results of anti-cancer effect between clinically used combinations and Ⅲ12 on MCF-7 cell line.

Predicted ranking	Group number	The IC50 (nM) of drug combinations				The best IC50
		Ⅰ	Ⅱ	Ⅲ	Ⅳ	
1	Ⅲ12	90.43	31.37	30.88	42.94	30.88
60	Ⅱ3	570.00	440.00	377.18	—	377.18
92	Ⅲ55	3695.00	7789.00	9444.00	814.10	814.10

Note: the predicted ranking included 120 drug combinations and individual drugs themselves.

#### 3.5.2 Synergistic effects of predicted combinations on triple-negative breast cancer cell line

Additionally, for the MDA-MB-468 cell line (triple-negative basal subtype), IC50 values for various drugs ranged from 881.6 nM to 536.2 μM, with paclitaxel exerting the greatest anti-cancer ability (Figure 4B). Then, the synergy impact of the top three combinations at various drug ratios was evaluated on MDA-MB-468. Similarly, Ⅲ12 (doxorubicin, docetaxel, and gemcitabine) achieved the best IC50 value of 115.5 nM when used in a 2:1:1 M ratio (Table 7). Additionally, the clinically used drug combinations Ⅲ55 (gemcitabine, epirubicin, and paclitaxel) (Telli and Carlson, 2009) was evaluated, and its best IC50 value was 774.2 nM, ranking 27th in the predicted results.

**TABLE 7:** The anti-cancer effects of drug combinations on MDA-MB-468 cells.

Predicted ranking	Group number	The IC50 (nM) of drug combinations				The best IC50
		Ⅰ	Ⅱ	Ⅲ	Ⅳ	
1	Ⅲ12	137.80	115.50	410.50	468.50	115.50
2	Ⅱ3	353.90	207.60	443.30	—	207.60
3	Ⅲ7	3497.00	522.90	1561.00	2767.00	522.90
27	Ⅲ55	3095.00	774.20	1056.00	1613.00	774.20

Note: the predicted ranking included 120 drug combinations and individual drugs themselves.

However, another triple-negative claudin-low subtype, MDA-MB-231, showed different drug responses. For example, monotherapy demonstrated that epirubicin had the lowest IC50 value of 2.81 μM while 5-fluorouracil remained the worst one (Figure 4C). Experiments indicated that IV33, a four-drug combination, was the best of the predicted top three. Two commonly used drug combinations (Ⅲ55 and Ⅱ3) in clinical treatment were also compared, and it was discovered that Ⅲ55, which was ranked 27th, and Ⅱ3, which was ranked 70th, had significantly higher IC50 values and inferior anticancer activity (Table 8).

**TABLE 8:** The anti-cancer effects of drug combinations on MDA-MB-231 cells.

Predicted ranking	Group number	The IC50 (nM) of drug combinations						The best IC50
		Ⅰ	Ⅱ	Ⅲ	Ⅳ	Ⅴ	Ⅵ	
1	Ⅳ33	177	52	139	2152	167	—	52
2	Ⅴ32	1087	743	868	852	219	552	219
3	Ⅳ59	147,600	1952	19,260	244	850	—	244
27	Ⅲ55	1506	5757	1793	5019	—	—	1506
70	Ⅱ3	7983	9414	4567	—	—	—	4567

Note: the predicted ranking included 120 drug combinations and individual drugs themselves.

In addition, the synergistic mechanisms of all combinations were calculated for MDA-MB-468 and MDA-MB-231, respectively. With regards to MDA-MB-468, all three top combinations indicated strong synergy at each drug ratio, with CI values smaller than 0.3. Besides, the clinically used Ⅲ55 showed strong synergy (CI = 0.27) and modest synergy (CI = 0.74) at 2:1:1 and 1:2:1 ratios, respectively; however, this regime had additive effect and antagonistic effect at the ratio of 1:1:2 (CI > 0.9) and 1:1:1 (CI > 1.1), respectively (Supplementary Figure S3). For the MDA-MB-231 cell line, the top two, IV33 and V32, exhibited strong synergistic effect at all drug ratios. However, the ranked third combination IV59 would exhibit some antagonistic activity at the ratio of 1:1:1:1 and 1:2:1:1, while still presenting strong synergy at other ratios. By contrast, II3 and III55, both of which have been used in clinical practice, had at least modest synergistic effects at each drug ratio (Supplementary Figure S4).

#### 3.5.3 Validation of predictive specificity for various breast cancer subtypes

The predicted result’s specificity for cancer cell lines was further confirmed. Ⅳ33, the best drug combination for MDA-MB-231, was evaluated using other subtypes of breast cancer cell lines such as MCF-7 and MDA-MB-468. Rather than that, Ⅲ12, which shown the greatest anticancer activity against MCF-7 and MDA-MB-468, was evaluated in a similar manner against MDA-MB-231. IV33 was predicted to rank 66th for MCF-7 and 33rd for MDA-MB-468, respectively, and Ⅲ12 was predicted to rank 10th for the MDA-MB-231 (Table 9). Experiments proved the anticancer abilities of various combinations predicted for each specific subtype. And regardless of the drug ratio, Ⅳ33 exhibited antagonistic activity against MCF-7; however, Ⅳ33 had synergistic anti-cancer effects on MDA-MB-468 at all but 1:1:1:1 and 1:1:1:2. Compared with the outcomes of IV33 on 2 cell lines, Ⅲ12, which performed slightly better on MDA-MB-231, exhibited synergy effect at all ratios.

**TABLE 9:** The anti-cancer effect of Ⅲ12 and Ⅳ33 on MCF-7, MDA-MB-231 and MDA-MB-468 cells.

Group number	Cell line	The IC50 (nM) of drug combinations					The best IC50
		Ⅰ	Ⅱ	Ⅲ	Ⅳ	Ⅴ	
Ⅲ12	MCF-7	90.4	31.3	30.8	42.9	—	30.8
	MDA-MB-468	137.8	115.5	410.5	468.5	—	115.5
	MDA-MB-231	15,720	8044.0	887.0	4666.0	—	887.0
Ⅳ33	MCF-7	8090.0	787.6	4355.0	788.9	1296.0	787.6
	MDA-MB-468	2803.0	1275.0	1988.0	1451.0	2665.0	1275.0
	MDA-MB-231	177.5	52.3	139.8	2152.0	167.5	52.3

To identify the potential synergistic mechanism of Ⅲ12 and Ⅳ33 on various breast cancer subtypes, KEGG pathway enrichment analysis was carried out with a *p*-value cutoff of 0.01 (Supplementary Figure S7). The enrichment analysis showed that pathway in cancer, PI3K-Akt signaling pathway and notch signaling pathway were the common pathways of Ⅲ12 and Ⅳ33 on three breast cancer subtypes. More importantly, MAPK signaling pathway may be a special mechanism for the synergistic anti-cancer effect of Ⅲ12 on MCF7 and MDA-MB-468, and Rap1 signaling pathway may be another important mechanism for Ⅲ12 on MCF7. In addition, MAPK signaling pathway was the common pathway of Ⅳ33 on three breast cancer subtypes. Further analysis revealed that Gap junction may not contribute significantly to the synergistic anti-cancer effect of Ⅳ33. Collectively, each subtype of breast cancer cell lines had its own best synergized drug combinations, indicating an excellent specificity of DeepMDS for cell line subtypes that reflect dramatic genetic and epigenetic changes during the development of cancer.

#### 3.5.4 Synergistic effects of predicted combinations on lung cancer cell line

An additional lung cancer cell line, A549, was used to examine the applicability of DeepMDS. It first predicted the synergistic anticancer effects of 120 drug combinations using DeepMDS (Supplementary Data Sheet D6), and then six combinations that ranked at the top two (Ⅱ28 and Ⅲ21), middle level (Ⅲ12 and Ⅲ47), or bottom two (Ⅲ41 and Ⅲ42) were examined in terms of cellular toxicity. IC50 values of individual drug ranged from 0.44 μM to 50.23 μM, with doxorubicin exerting the best anti-cancer ability and 5-fluorouracil still having the weakest efficacy (Figure 4D). It was shown that the anti-cancer capacity of various combinations at their optimal drug ratios was compatible with the predicted results of DeepMDS, thereby proving the reliability of this model for predicting potential multi-drug combinations (Table 10).

**TABLE 10:** The anti-cancer effects of drug combinations on A549 cells.

Predicted ranking	Group number	The IC50 (nM) of drug combinations				The best IC50
		Ⅰ	Ⅱ	Ⅲ	Ⅳ	
1	Ⅱ28	181.5	140.9	73.8	—	73.8
2	Ⅲ21	240.6	194.2	240.3	234.1	194.2
30	Ⅲ12	215.1	135.4	128.4	191.1	128.4
41	Ⅲ47	1353.0	696.8	750.7	3213.0	696.8
119	Ⅲ41	9304	15,100	4739	15,110	4739
125	Ⅲ42	1353	3807	1112	1223	1112

Note: the predicted ranking included 120 drug combinations and individual drugs themselves.

When we looked at the mechanisms of action for these combinations, it was clear that the synergy effect at every drug ratio was the most noticeable benefit of the top two combinations. Combinations in the middle (III47), as well as those at the bottom, showed additive or antagonistic effects at the majority of the ratios, but the 30th-ranked combination (III12) also demonstrated a strong synergistic mechanism in some cases (Supplementary Figure S6). It is possible that some underlying drug-target interactions, which were part of the overall synergistic mechanism, were not collected in the current training dataset because the predicted rank for Ⅲ12 and Ⅲ42 differed from the experimental results. Big biomedical data, which is becoming more widely available, could help us better predict the best combination for a given cancer cell line.

## 4 Discussion

Although it is now well established that combination therapies are significantly more effective at treating complicated disorders, experimentally assessing novel combinations is difficult due to the huge number of possible drug combinations. In this study, a deep learning-based model (DeepMDS) was successfully built to expedite the development of novel synergistic multi-drug combinations for clinical cancer treatment. DeepMDS enabled the ranking of all multi-drug combinations constructed randomly from a pool of medications using large-scale integrated features taken from gene expression profiles of human cancer cell lines, the multiscale interactome, and drug response data. Also, the predicted ranking of drug combinations revealed the likely mechanisms of action; for example, the higher ranked combination had a significantly greater synergy impact, whereas the lower ranked combination would have an antagonistic effect. DeepMDS performed admirably in terms of accuracy. For the classification job, it earned an ACC of 0.94, an AUC of 0.97, a SEN of 0.95, and a SPE of 0.93. When facing a regression task, this model achieved a MSE of 2.50 and a RMSE of 1.58. A lack of experimental validation for some deep learning-based models may result in erroneous and/or unprofitable predictions when evaluating combinations of unknown druggable chemicals, natural products, and/or new cell lines. So, an *in vitro* cell experiment with seven clinically used anti-cancer drugs was used to test the ranked drug combinations predicted by DeepMDS in this work. In comparison to other drug combinations, it is clear that all of the predicted optimal synergistic combinations had a significant synergistic anti-cancer effect on each individual cell line.

As per the knowledge of authors, one of the biggest advantages of our model is to accurately predict the most promising three- or more-drug combinations for a certain cancer cell line. High-order combinations of drugs, as opposed to simple drug pairs, can regulate many anti-cancer networks simultaneously, hence improving tumor growth inhibition efficacy while avoiding drug resistance. Results indicated that DeepMDS leveraged its ability to rank high-order combinations which were randomly formed in the training space and so far untested. In addition, another advantage of DeepMDS is to predict synergistic combinations specific to a cancer cell line and even to a subtype of cell line. Experiments demonstrated that DeepMDS consistently gained high prediction performance across various subtypes of breast cancer cells and tissue-specific cancer cell lines. For example, III12 (doxorubicin, docetaxel and gemcitabine) had the best synergistic anti-cancer activity on hormone-responsive breast cancer cell line MCF-7, but Ⅳ33 (doxorubicin, gemcitabine, methotrexate and paclitaxel) and Ⅲ12 were the most effective combinations against claudin-low MDA-MB-231 and basal MDA-MB-468, respectively, for triple-negative breast cancers. A549, a lung cancer cell line, was also used to evaluate the cell line specificity of DeepMDS, and one drug pair (Ⅱ28) was found to be the best regimen. Therefore, it doesn’t matter what the multi-drug combinations are, DeepMDS was able to accurately predict and rank synergistic combinations against the cell line of interest, showing a wide range of applications. DeepMDS, in particular, makes it easier in the future to give targeted multi-drug combinations when taking into account the heterogeneity in genomic data of each patient.

To further validate the prediction power of DeepMDS for unseen combinations, clinically used breast cancer drug combinations, including II3 (doxorubicin and docetaxel) and Ⅲ55 (gemcitabine, epirubicin, and paclitaxel), were tested in three subtypes of breast cancer cell line and, by extension, compared with the predicted best combinations. As a result, the IC50 values of Ⅱ3 and Ⅲ55 against MCF-7 increased about tenfold to twentyfold when compared to the predicted best combination (III12). Also, for triple-negative MDA-MB-468, this triple-drug combination (III12) was predicted to be the best, with an IC50 value of approximately 55.6 percent of II3’s and 14.9 percent of III55’s, respectively. In another triple-negative MDA-MB-231, we observed that the IC50 values of II3 leaped by about 88-fold, of III55 by 28.9-fold, when compared to the best combination IV33. Thus, it is possible to apply the novel synergistic drug combinations predicted by DeepMDS for breast cancer clinical trials, especially with regard to the triple negative breast cancer.

During the construction of machine learning and deep learning models, data are of critical relevance. In some cases, low quality predictive performance was mainly due to the incomplete dataset. For example, DeepSynergy was unable to accurately predict the response of novel medications and novel cell lines; more specifically, DeepSynergy indicated MSEs between 414 and 500 for novel drugs, and MSEs between 387 and 461 for novel cell lines. Because there were only 38 training instances of chemical compounds and cell lines, the authors speculated that the low prediction performance was due to a lack of training data (39 examples). In this case, the larger-scale integrated modeling datasets (201,405), which include 1,000 human cancer cell lines and 1839 chemicals, could substantially improve the performance and increase the accuracy of ranking the combinations. Another characteristic is the incorporation of drug-target data into modeling data. Rather than relying on descriptors of chemical structures to compare the structure similarity of two drugs, the drug-target information drives our prediction model to produce more accurate results in a biomedical context, which is beneficial for elucidating the underlying mechanisms of synergy action.

However, one limitation of our suggested strategy is that the modeling data contains insufficient information on drug targets. As a result, in some situations, a portion of a drug’s target information may be omitted from the existing features, resulting in a discrepancy between predicted and actual outcomes. Notably, we did not feel that this constraint would eliminate the clinical use of our DeepMDS. By updating experimental drug targets data or adding predicted drug targets, this problem can be solved and the prediction accuracy of DeepMDS can be further enhanced. In addition, drug concentration ratio is also important for the synergistic effect of drug combination. Due to the lack of available data on drug concentration ratios of drug combinations and the corresponding synergies, in this model, it was assumed that the drug concentration of each drug was sufficient to act on their targets and produce efficacy. We will continue to develop a computational method to predict the optimal drug concentration ratio for drug combination in future studies. Despite the limitations, our prediction model was able to translate monotherapy data into clinically useful predictions and expand the Universe of possible synergistic medication combinations, prioritizing promising multi-drug combinations for distinct types of cancer.

## 5 Conclusion

In this study, we developed a deep learning-based model that could aid in the discovery of the probable best combinations for a certain cell line or cell subtype. With regard to the high-cost experimental screening of drug combinations, our DeepMDS would significantly simplify the process of prioritizing the most promising multi-drug combinations for future pre-clinical studies. More importantly, our experimental validation proved that high-order combinations including three or more drugs, in most of cases, consistently outperformed drug pairs typically utilized in clinical treatment. Also, precise and robust prediction of drug combinations could identify the possible targeted combinations for personalized medicine, thereby expediting the development of combination therapy to combat against drug resistance and to improve efficacy.

## Data Availability

The datasets presented in this study can be found in online repositories. The names of the repository/repositories and accession number(s) can be found in the article/supplementary material.

## References

[B1] Alexander S. Friedl P. (2012). Cancer invasion and resistance: Interconnected processes of disease progression and therapy failure. Trends Mol. Med. 18 (1), 13–26. 10.1016/j.molmed.2011.11.003 22177734

[B2] Aliper A. Plis S. Artemov A. Ulloa A. Mamoshina P. Zhavoronkov A. (2016). Deep learning applications for predicting pharmacological properties of drugs and drug repurposing using transcriptomic data. Mol. Pharm. 13 (7), 2524–2530. 10.1021/acs.molpharmaceut.6b00248 27200455 PMC4965264

[B3] Bansal M. Yang J. Karan C. Menden M. P. Costello J. C. Tang H. (2014). A community computational challenge to predict the activity of pairs of compounds. Nat. Biotechnol. 32 (12), 1213–1222. 10.1038/nbt.3052 25419740 PMC4399794

[B4] Cerami E. Gao J. Dogrusoz U. Gross B. E. Sumer S. O. Aksoy B. A. (2012). The cBio cancer genomics portal: An open platform for exploring multidimensional cancer genomics data. Cancer Discov. 2 (5), 401–404. 10.1158/2159-8290.Cd-12-0095 22588877 PMC3956037

[B5] Chen G. Tsoi A. Xu H. Zheng W. J. (2018). Predict effective drug combination by deep belief network and ontology fingerprints. J. Biomed. Inf. 85, 149–154. 10.1016/j.jbi.2018.07.024 30081101

[B6] Chou T.-C. Martin N. (2007). strong>The mass-action law-based new computer software, CompuSyn, for automated simulation of synergism and antagonism in drug combination studies</strong&gt. Cancer Res. 67 (9), 637.

[B7] Chou T. C. Talalay P. (1984). Quantitative analysis of dose-effect relationships: The combined effects of multiple drugs or enzyme inhibitors. Adv. Enzyme Regul. 22, 27–55. 10.1016/0065-2571(84)90007-4 6382953

[B8] Davies G. Boeree M. Hermann D. Hoelscher M. (2019). Accelerating the transition of new tuberculosis drug combinations from Phase II to Phase III trials: New technologies and innovative designs. PLoS Med. 16 (7), e1002851. 10.1371/journal.pmed.1002851 31287813 PMC6615592

[B9] Dry J. R. Yang M. Saez-Rodriguez J. (2016). Looking beyond the cancer cell for effective drug combinations. Genome Med. 8 (1), 125. 10.1186/s13073-016-0379-8 27887656 PMC5124246

[B10] Feng C. Chen H. Yuan X. Sun M. Chu K. Liu H. (2019). Gene expression data based deep learning model for accurate prediction of drug-induced liver injury in advance. J. Chem. Inf. Model. 59 (7), 3240–3250. 10.1021/acs.jcim.9b00143 31188585

[B11] Gayvert K. M. Aly O. Platt J. Bosenberg M. W. Stern D. F. Elemento O. (2017). A computational approach for identifying synergistic drug combinations. PLoS Comput. Biol. 13 (1), e1005308. 10.1371/journal.pcbi.1005308 28085880 PMC5234777

[B12] Gentleman R. C. Carey V. J. Bates D. M. Bolstad B. Dettling M. Dudoit S. (2004). Bioconductor: Open software development for computational biology and bioinformatics. Genome Biol. 5 (10), R80. 10.1186/gb-2004-5-10-r80 15461798 PMC545600

[B13] Gotwals P. Cameron S. Cipolletta D. Cremasco V. Crystal A. Hewes B. (2017). Prospects for combining targeted and conventional cancer therapy with immunotherapy. Nat. Rev. Cancer 17 (5), 286–301. 10.1038/nrc.2017.17 28338065

[B14] Holliday D. L. Speirs V. (2011). Choosing the right cell line for breast cancer research. Breast Cancer Res. 13 (4), 215. 10.1186/bcr2889 21884641 PMC3236329

[B15] Holohan C. Van Schaeybroeck S. Longley D. B. Johnston P. G. (2013). Cancer drug resistance: An evolving paradigm. Nat. Rev. Cancer 13 (10), 714–726. 10.1038/nrc3599 24060863

[B16] Huang L. Brunell D. Stephan C. Mancuso J. Yu X. He B. (2019). Driver network as a biomarker: Systematic integration and network modeling of multi-omics data to derive driver signaling pathways for drug combination prediction. Bioinformatics 35 (19), 3709–3717. 10.1093/bioinformatics/btz109 30768150 PMC6761967

[B17] Ianevski A. Giri A. K. Aittokallio T. (2020). SynergyFinder 2.0: Visual analytics of multi-drug combination synergies. Nucleic Acids Res. 48 (1), W488–W493. 10.1093/nar/gkaa216 32246720 PMC7319457

[B18] Iorio F. Knijnenburg T. A. Vis D. J. Bignell G. R. Menden M. P. Schubert M. (2016). A landscape of pharmacogenomic interactions in cancer. Cell. 166 (3), 740–754. 10.1016/j.cell.2016.06.017 27397505 PMC4967469

[B19] Iwata H. Sawada R. Mizutani S. Kotera M. Yamanishi Y. (2015). Large-scale prediction of beneficial drug combinations using drug efficacy and target profiles. J. Chem. Inf. Model. 55 (12), 2705–2716. 10.1021/acs.jcim.5b00444 26624799

[B20] Jeon M. Kim S. Park S. Lee H. Kang J. (2018). *In silico* drug combination discovery for personalized cancer therapy. BMC Syst. Biol. 12 (2), 16. 10.1186/s12918-018-0546-1 29560824 PMC5861486

[B21] Kuru H. b. Tastan O. Cicek E. (2021). MatchMaker: A deep learning framework for drug synergy prediction. IEEE/ACM Trans. Comput. Biol. Bioinform. 19, 2334–2344. 10.1109/TCBB.2021.3086702 34086576

[B22] MacGowan A. P. Holt H. A. Reeves D. S. (1990). *In-vitro* synergy testing of nine antimicrobial combinations against Listeria monocytogenes. J. Antimicrob. Chemother. 25 (4), 561–566. 10.1093/jac/25.4.561 2112538

[B23] Mahase E. (2019). Breast cancer: NICE approves new drug combination treatment. Bmj 366, l4727. 10.1136/bmj.l4727 31315824

[B24] Menden M. P. Wang D. Mason M. J. Szalai B. Bulusu K. C. Guan Y. (2019). Community assessment to advance computational prediction of cancer drug combinations in a pharmacogenomic screen. Nat. Commun. 10 (1), 2674. 10.1038/s41467-019-09799-2 31209238 PMC6572829

[B25] National Comprehensive Cancer Network (2021). Breast cancer. Version 5.2021. Available: www.nccn.org/professionals/physician_gls/pdf/breast.pdf (Accessed June 14, 2021).

[B26] Parkinson H. Sarkans U. Shojatalab M. Abeygunawardena N. Contrino S. Coulson R. (2005). ArrayExpress--a public repository for microarray gene expression data at the EBI. Nucleic Acids Res. 33, D553–D555. Database issue). 10.1093/nar/gki056 15608260 PMC540010

[B27] Preuer K. Lewis R. P. I. Hochreiter S. Bender A. Bulusu K. C. Klambauer G. (2018). DeepSynergy: Predicting anti-cancer drug synergy with deep learning. Bioinformatics 34 (9), 1538–1546. 10.1093/bioinformatics/btx806 29253077 PMC5925774

[B28] Ru J. Li P. Wang J. Zhou W. Li B. Huang C. (2014). Tcmsp: A database of systems pharmacology for drug discovery from herbal medicines. J. Cheminform. 6, 13. 10.1186/1758-2946-6-13 24735618 PMC4001360

[B29] Sidorov P. Naulaerts S. Ariey-Bonnet J. Pasquier E. Ballester P. J. (2019). Predicting synergism of cancer drug combinations using NCI-ALMANAC data. Front. Chem. 7, 509. 10.3389/fchem.2019.00509 31380352 PMC6646421

[B30] Sopirala M. M. Mangino J. E. Gebreyes W. A. Biller B. Bannerman T. Balada-Llasat J. M. (2010). Synergy testing by Etest, microdilution checkerboard, and time-kill methods for pan-drug-resistant Acinetobacter baumannii. Antimicrob. Agents Chemother. 54 (11), 4678–4683. 10.1128/aac.00497-10 20713678 PMC2976112

[B31] Sun W. Sanderson P. E. Zheng W. (2016). Drug combination therapy increases successful drug repositioning. Drug Discov. Today 21 (7), 1189–1195. 10.1016/j.drudis.2016.05.015 27240777 PMC4907866

[B32] Sun X. Vilar S. Tatonetti N. P. (2013). High-throughput methods for combinatorial drug discovery. Sci. Transl. Med. 5 (205), 205rv1. 205rv201. 10.1126/scitranslmed.3006667 24089409

[B33] Sun Y. Sheng Z. Ma C. Tang K. Zhu R. Wu Z. (2015). Combining genomic and network characteristics for extended capability in predicting synergistic drugs for cancer. Nat. Commun. 6, 8481. 10.1038/ncomms9481 26412466 PMC4598846

[B34] Telli M. L. Carlson R. W. (2009). First-line chemotherapy for metastatic breast cancer. Clin. Breast Cancer 9 (2), S66–S72. 10.3816/CBC.2009.s.007 19596645

[B35] Tolomeo M. Simoni D. (2002). Drug resistance and apoptosis in cancer treatment: Development of new apoptosis-inducing agents active in drug resistant malignancies. Curr. Med. Chem. Anticancer. Agents 2 (3), 387–401. 10.2174/1568011024606361 12678739

[B36] Wang Y. Xiao J. Suzek T. O. Zhang J. Wang J. Bryant S. H. (2009). PubChem: A public information system for analyzing bioactivities of small molecules. Nucleic Acids Res. 37, W623–W633. 10.1093/nar/gkp456 19498078 PMC2703903

[B37] Wiesner J. Henschker D. Hutchinson D. B. Beck E. Jomaa H. (2002). *In vitro* and *in vivo* synergy of fosmidomycin, a novel antimalarial drug, with clindamycin. Antimicrob. Agents Chemother. 46 (9), 2889–2894. 10.1128/aac.46.9.2889-2894.2002 12183243 PMC127394

[B38] Wishart D. S. Feunang Y. D. Guo A. C. Lo E. J. Marcu A. Grant J. R. (2018). DrugBank 5.0: A major update to the DrugBank database for 2018. Nucleic Acids Res. 46 (D1), D1074–d1082. 10.1093/nar/gkx1037 29126136 PMC5753335

[B39] Zhang H. Feng J. Zeng A. Payne P. Li F. (2021a). Predicting tumor cell response to synergistic drug combinations using a novel simplified deep learning model. AMIA Annu. Symp. Proc. 2020, 1364–1372. 33936513 PMC8075535

[B40] Zhang T. Zhang L. Payne P. R. O. Li F. (2021b). Synergistic drug combination prediction by integrating Multiomics data in deep learning models. Methods Mol. Biol. 2194, 223–238. 10.1007/978-1-0716-0849-4_12 32926369

