# Supplementary material for: Deep learning-based multi-drug synergy prediction model for individually tailored anti-cancer therapies
Source: Front Pharmacol. 2022 Dec 15;13:1032875. doi: 10.3389/fphar.2022.1032875 (PMC9797718; doi:10.3389/fphar.2022.1032875)
Supplement: Supplementary file 1 [file DataSheet1.docx]

Supplementary Material

# Supplementary Figures and Tables

## Supplementary Figures


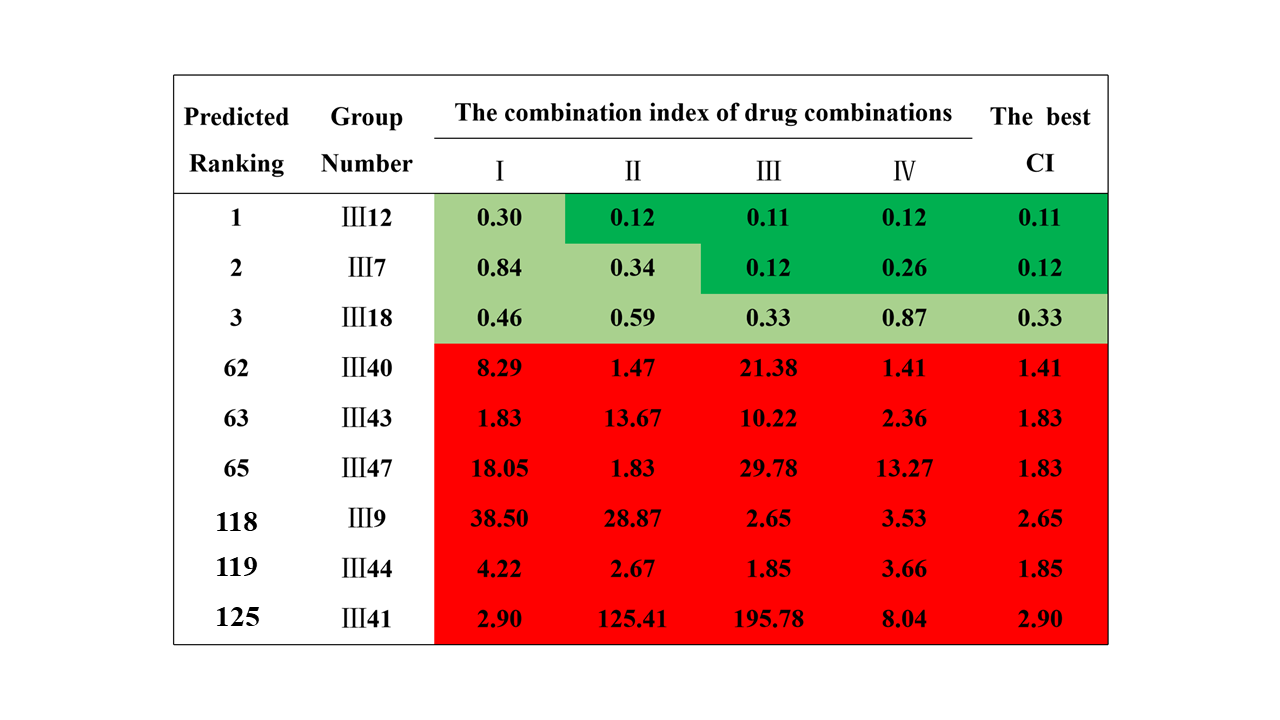


**Supplementary Figure S1.** The combination index of nine combinations of three drugs on MCF-7 cells

**
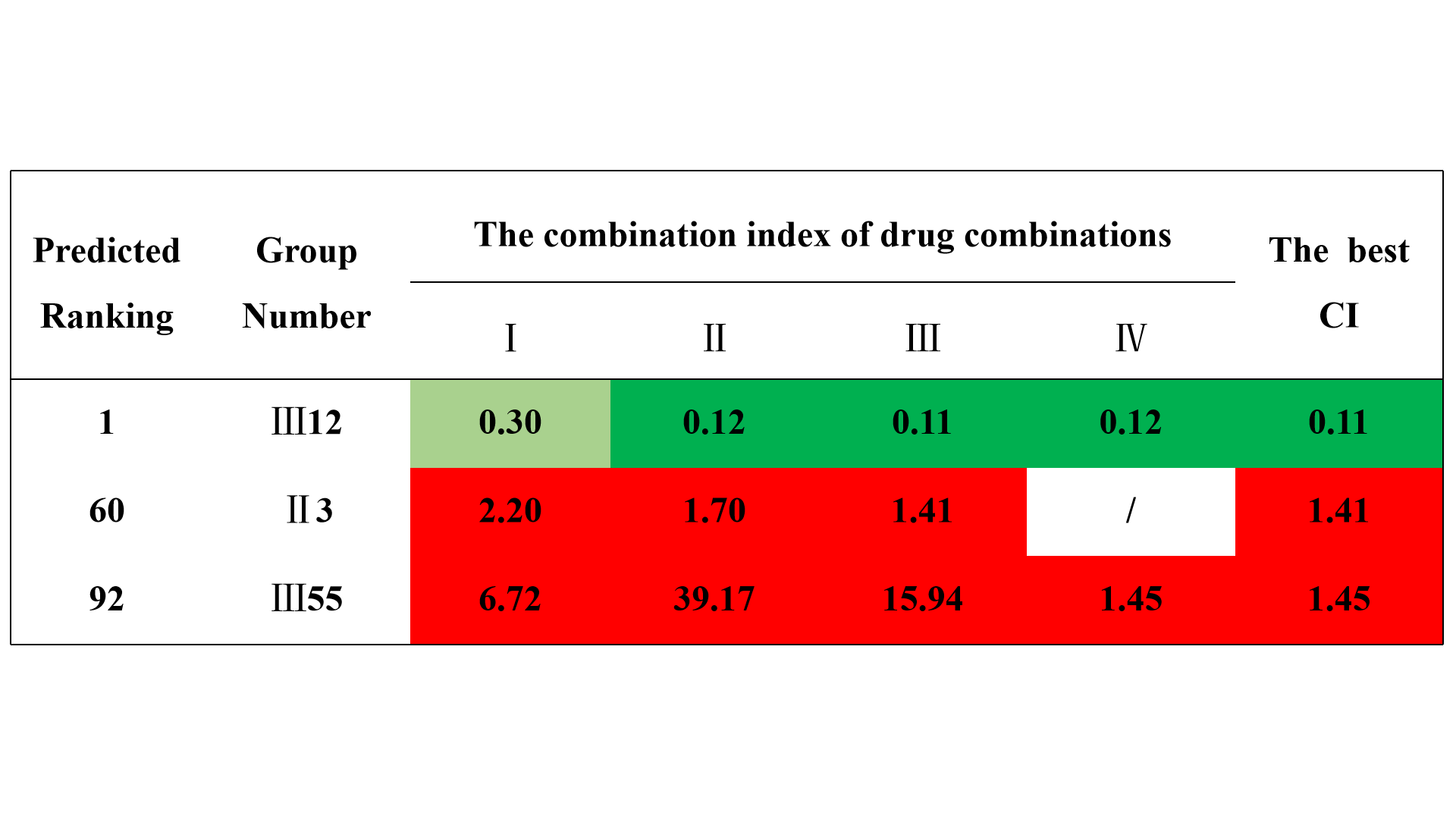
**

**Supplementary Figure S2.** The combination index of clinically used combinations and Ⅲ12 on MCF-7 cells


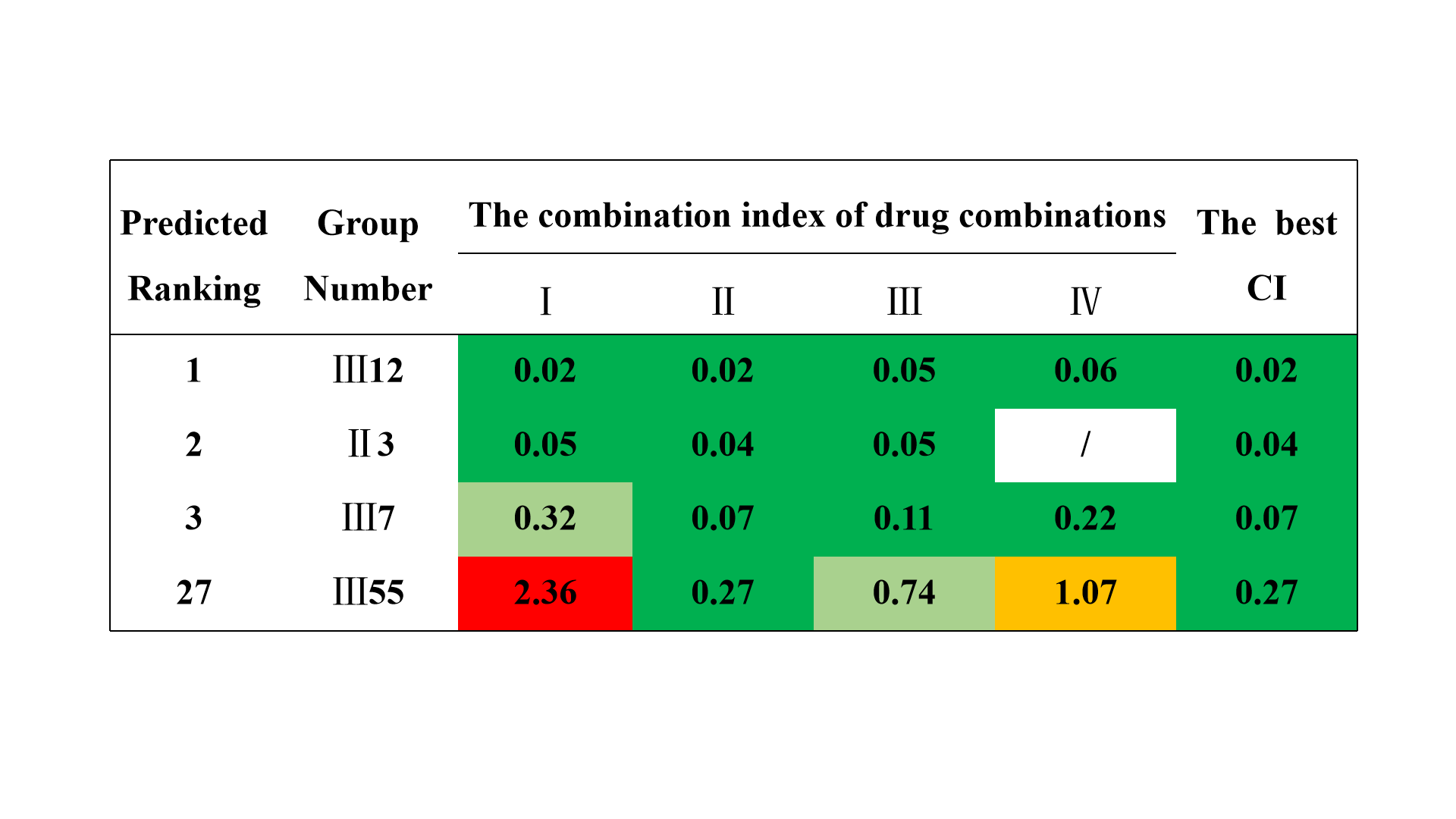


**Supplementary Figure S3.** The combination index of drug combinations on MDA-MB-468


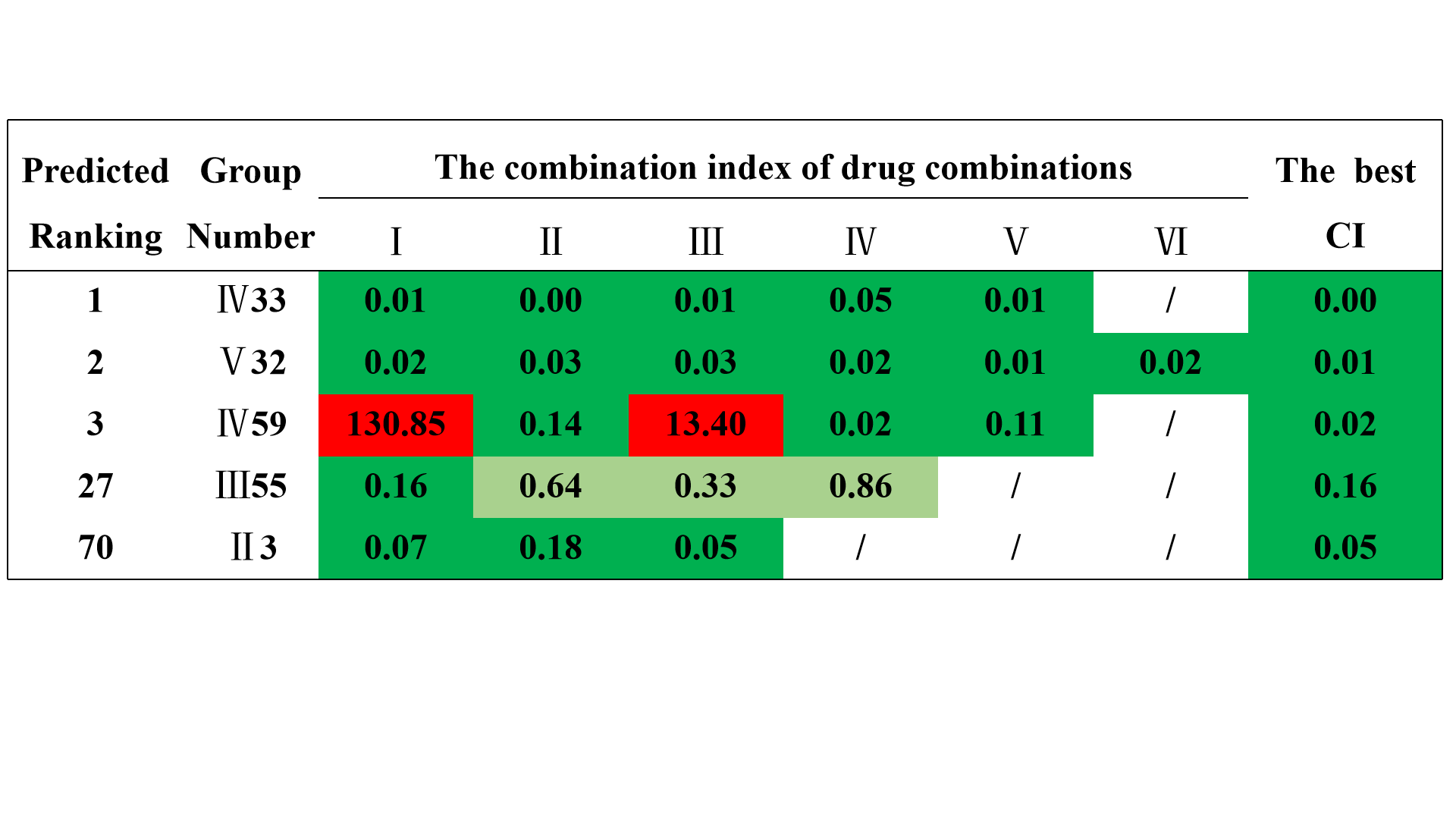


**Supplementary Figure S4.** The combination index of drug combinations on MDA-MB-231


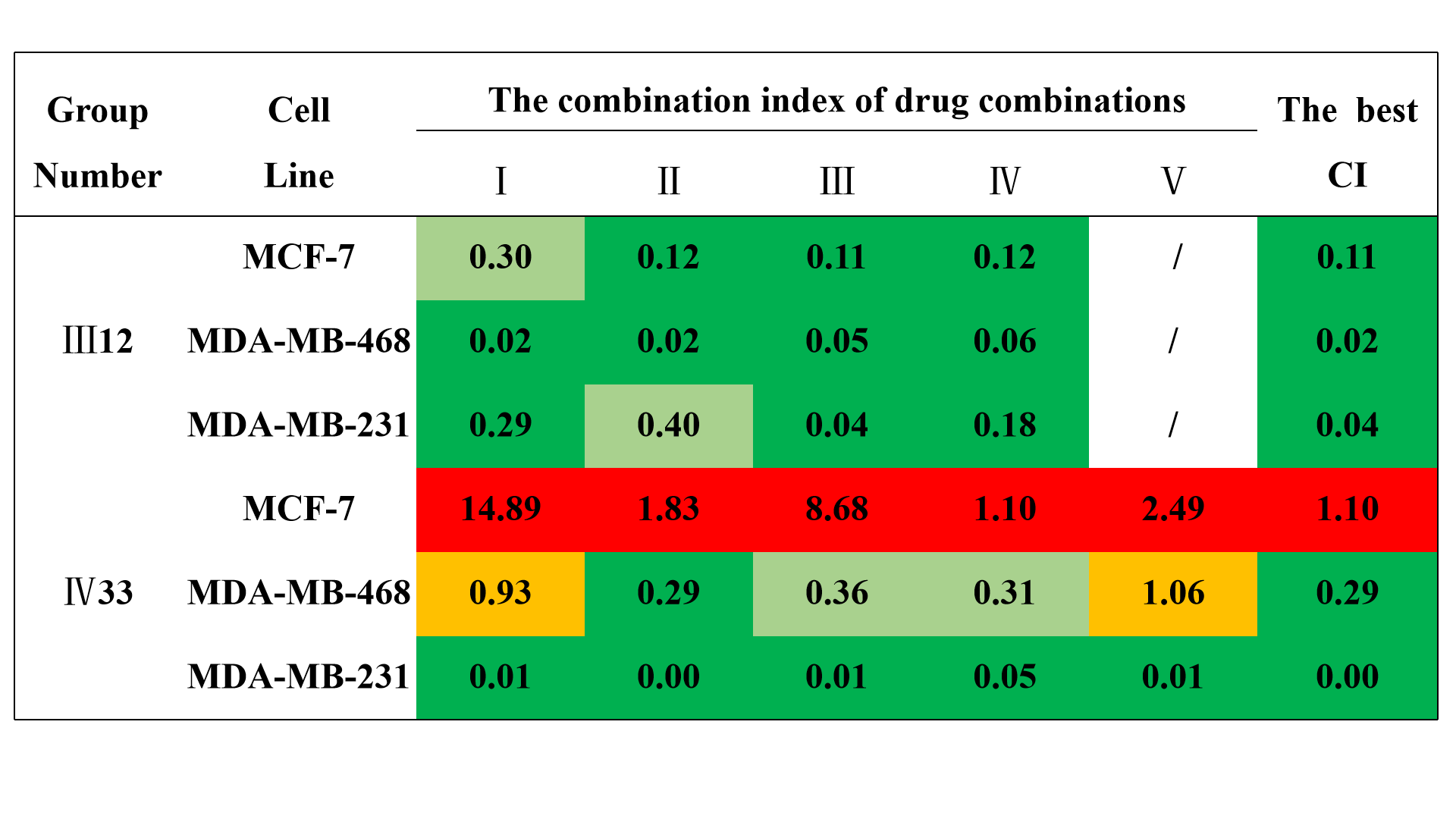
 **Supplementary Figure S5.** The combination index of Ⅲ12 and Ⅳ33 on MCF-7, MDA-MB-231 and MDA-MB-468 cells


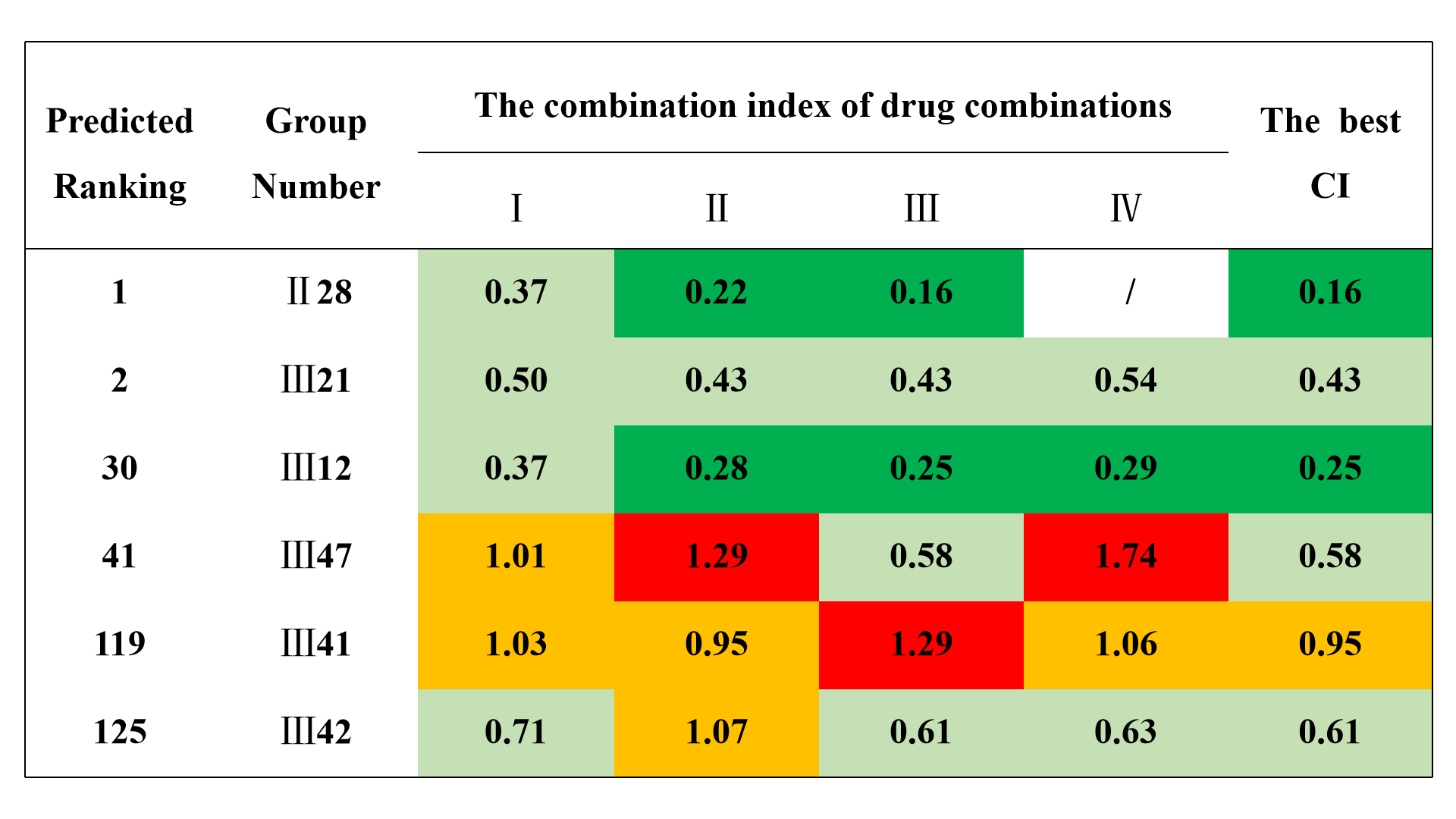


**Supplementary Figure S6.** The combination index of drug combinations on A549 cells.


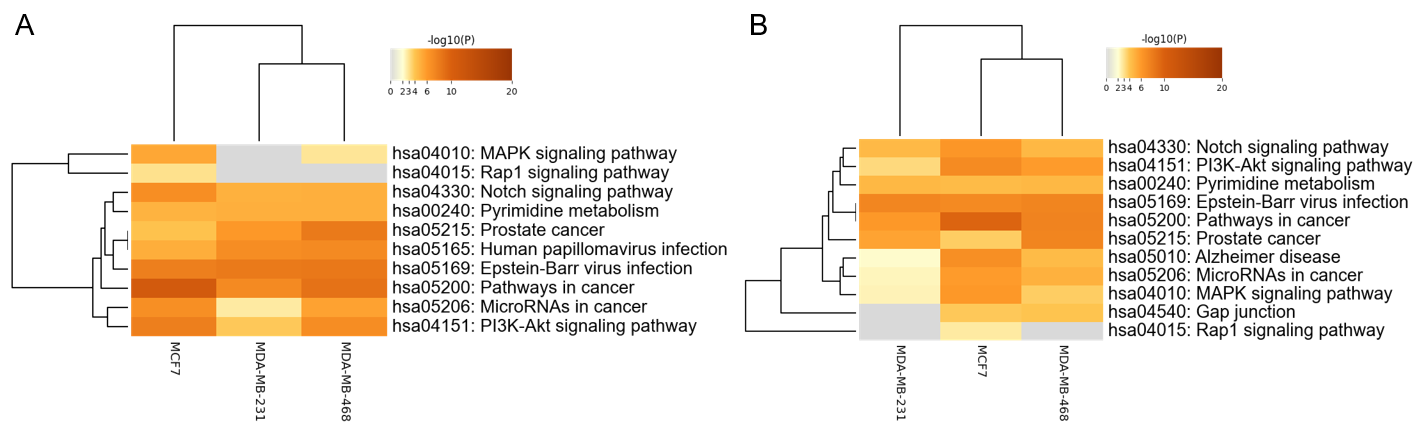


**Supplementary Figure S7.** KEGG pathway enrichment analysis of Ⅲ12 and Ⅳ33 on three breast cancer subtypes.

## Supplementary Tables

**Supplementary Table S1.** Comparison of DeepMDS performance to predictive models from previous literature

| Year | Study name | Correlation | Ref |
| --- | --- | --- | --- |
|  | DeepMDS (Our study) | 0.93 (p) |  |
| 2018 | DeepSynergy | 0.73 (p) | (Preuer et al., 2018) |
| 2020 | Deep graph | 0.71 for breast (p)  0.67 for lung (p) | (Jiang et al., 2020) |
| 2020 | comboFM | 0.72 (p) | (Julkunen et al., 2020) |
| 2021 | DrugCell | 0.8 (s) | (Kuenzi et al., 2020) |
| 2021 | AuDNNsynergy | 0.74 (p) | (Zhang et al., 2021) |

Note: p is Pearson correlation coefficient, and s is Spearman's correlation coefficient.

**Supplementary Table S2**. Parameters for deep learning prediction model optimization

| Parameters | Value space for optimization | Optimal value of parameters | |
| --- | --- | --- | --- |
|  |  | Regression | Classification |
| Epoch number | 10, 50, 100, 200, 500 | 200 | 500 |
| Batch size | 10, 32, 64, 128, 256 | 128 | 32 |
| Learning rate | 0.01, 0.001, 0.00001 | 0.00001 | 0.001 |
| Dropout rate | 0, 0.2, 0.5 | 0.5 | 0.5 |
| Hidden units_1st | 50, 100, 200, 500, 1000 | 200 | 200 |
| Hidden units_2st | 50, 100, 200, 500, 1000 | 100 | 100 |

**Supplementary Table S3.** Parameters for KNN prediction model optimization.

| Parameters | Value space for optimization | Optimal value of parameters | |
| --- | --- | --- | --- |
|  |  | Regression | Classification |
| Neighbors numbers | 1, 2, 3, 4, 5, 6, 7, 8, 9, 10 | 6 | 5 |
| Weight functions | ‘uniform’, ‘distance’ | ‘uniform’ | ‘uniform’ |
| Algorithm | ‘ball_tree’,‘kd_tree’,‘brute’,‘auto’ | ‘auto’ | ‘auto’ |

**Supplementary Table S4.** Parameters for RF prediction model optimization.

| Parameters | Value space for optimization | Optimal value of parameters | |
| --- | --- | --- | --- |
|  |  | Regression | Classification |
| Estimators number | 50, 100, 200, 500, 1000 | 200 | 100 |
| Features considered | ‘log2’, ‘sqrt’, ‘auto’ | ‘auto’ | ‘auto’ |
| Min_samples_leaf | 1, 5, 10, 50, 100, 200 | 50 | 10 |

**Supplementary Table S5.** Parameters for SVM prediction model optimization.

| Parameters | Value space for optimization | Optimal value of parameters | |
| --- | --- | --- | --- |
|  |  | Regression | Classification |
| kernel function | Linear, Polynomial, RBF, Sigmoid | RBF | RBF |
| C | 0.1, 1, 10, 100, 1000 | 10 | 1 |
| gamma | 0.001, 0.01, 0.1, 1, 10 | 0.01 | 0.1 |

**Supplementary Table S6.** Parameters for GBM prediction model optimization.

| Parameters | Value space for optimization | Optimal value of parameters | |
| --- | --- | --- | --- |
|  |  | Regression | Classification |
| Estimators number | 50, 100, 200, 500, 1000 | 500 | 200 |
| min_ samples_ split | 200, 400, 600, 800, 1000, 2000 | 1000 | 600 |
| Learning rate | 0.1, 0.01, 0.001 | 0.01 | 0.01 |
| min_ samples_ leaf | 30, 40, 50, 60, 70 | 60 | 60 |

**References**

Jiang, P., Huang, S., Fu, Z., Sun, Z., Lakowski, T.M., and Hu, P. (2020). Deep graph embedding for prioritizing synergistic anticancer drug combinations. *Comput Struct Biotechnol J* 18**,** 427-438. doi: 10.1016/j.csbj.2020.02.006.

Julkunen, H., Cichonska, A., Gautam, P., Szedmak, S., Douat, J., Pahikkala, T., et al. (2020). Leveraging multi-way interactions for systematic prediction of pre-clinical drug combination effects. *Nat Commun* 11(1)**,** 6136. doi: 10.1038/s41467-020-19950-z.

Kuenzi, B.M., Park, J., Fong, S.H., Sanchez, K.S., Lee, J., Kreisberg, J.F., et al. (2020). Predicting Drug Response and Synergy Using a Deep Learning Model of Human Cancer Cells. *Cancer Cell* 38(5)**,** 672-684.e676. doi: 10.1016/j.ccell.2020.09.014.

Preuer, K., Lewis, R.P.I., Hochreiter, S., Bender, A., Bulusu, K.C., and Klambauer, G. (2018). DeepSynergy: predicting anti-cancer drug synergy with Deep Learning. *Bioinformatics* 34(9)**,** 1538-1546. doi: 10.1093/bioinformatics/btx806.

Zhang, T., Zhang, L., Payne, P.R.O., and Li, F. (2021). Synergistic Drug Combination Prediction by Integrating Multiomics Data in Deep Learning Models. *Methods Mol Biol* 2194**,** 223-238. doi: 10.1007/978-1-0716-0849-4_12.
